# Single‐Cell and Spatial Omics: Methods and Applications

**DOI:** 10.1002/mco2.70713

**Published:** 2026-04-06

**Authors:** Xiaoping Cen, Xiaolan Huang, Enjin Deng, Xue Gong, Na Tan, Jifeng Ye, Yin Wang, Roland Eils, Qun Luo, Yixue Li, Fangfang Qu

**Affiliations:** ^1^ Guangzhou National Laboratory, Guangzhou International Bio Island Guangzhou Guangdong Province China; ^2^ State Key Laboratory of Oncology in South China Collaborative Innovation Center for Cancer Medicine Sun Yat‐sen University Cancer Center Guangzhou China; ^3^ Zhongshan School of Medicine Sun Yat‐Sen University Guangzhou China; ^4^ College of Animal Science and Technology Guangxi University Nanning Guangxi China; ^5^ GMU‐GIBH Joint School of Life Sciences Guangdong Provincial Key Laboratory of Protein Modification and Disease The Guangdong‐Hong Kong‐Macao Joint Laboratory for Cell Fate Regulation and Diseases Guangzhou Medical University Guangzhou China; ^6^ Department of Biomedical Engineering School of Intelligent Medicine China Medical University Shenyang Liaoning China; ^7^ Digital Health Center Berlin Institute of Health (BIH) Charité ‐ Universitätsmedizin Berlin Berlin Germany; ^8^ Intelligent Medicine Institute Fudan University Shanghai China; ^9^ State Key Laboratory of Respiratory Disease the First Affiliated Hospital, Guangzhou Medical University Guangzhou Guangdong China; ^10^ Key Laboratory of Systems Health Science of Zhejiang Province School of Life Science Hangzhou Institute for Advanced Study University of Chinese Academy of Sciences Hangzhou China; ^11^ School of Life Sciences and Biotechnology Shanghai Jiao Tong University Shanghai China; ^12^ Shanghai Institute of Nutrition and Health Chinese Academy of Sciences Shanghai China

**Keywords:** artificial intelligence, foundation models, multi‐omics integration, precision medicine, single‐cell omics, spatial omics

## Abstract

Single‐cell and spatial omics have revolutionized biomedical research by enabling high‐resolution molecular profiling across cells and tissues, thereby overcoming key limitations of bulk sequencing and revealing unprecedented cellular heterogeneity and spatial organization central to development, homeostasis, and disease. Specifically, advances in high‐throughput, subcellular, and multiomics profiling are promoting the field toward deeper insights. In parallel, computational progress, including generative artificial intelligence (AI) and foundation models, is developing rapidly for manipulating multimodal multiomics data. These advancements have been applied to diverse diseases and biological systems, facilitating innovative biomedical findings. However, a significant gap persists between rapid methodological advances and their systematic application for deciphering human biology and pathology. This review synthesizes recent breakthroughs in single‐cell and spatial technologies and surveys computational methods, including AI‐driven approaches, foundation models, and multi‐omics integration algorithms for both single‐cell and spatial analyses. We then summarize representative applications across major human organ systems in health and disease, highlighting opportunities for biomarker discovery, therapeutic target identification, and precision medicine. Finally, we discuss current challenges and future directions for bridging technological innovation with robust biomedical discovery and translational impact. This review provides a vital guide for researchers in the field, offering critical insights for accelerating the translation of single‐cell and spatial omics.

## Graphical abstract

- Systematically summarized the breakthrough sequencing technologies and computational methods for single‐cell and spatial omics across multiple omics layers, including genome, epigenome, transcriptome, proteome, and metabolome.
- State‐of‐the‐art methods for multi‐omics integration, cross‐modal integration, and cross‐scale integration were reviewed, with AI‐based algorithms and foundation models were highlighted.
- Comprehensively summarized the application of single‐cell and spatial omics in human biology and diseases of all organs, identifying how single‐cell and spatial omics drive biomedical discovery.
- Critically discussed current challenges and future directions for the field, especially how to fill the gap between methodological developments and applications.

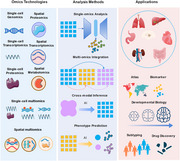

## 1 Introduction

Single‐cell and spatial omics technologies have transformed the study of human biology by enabling molecular profiling at single‐cell resolution and within intact tissue architecture. Unlike conventional bulk sequencing techniques that measure averaged signals across heterogeneous populations, single‐cell approaches characterize distinct cell types, transitional states, lineage relationships, and regulatory programs across the genome, epigenome, transcriptome, proteome, and metabolome [1]. Spatial omics technologies further address the limitations of single‐cell sequencing by integrating molecular profiling with spatial localization, providing a map for molecular data directed to tissue architectures [2]. Together, these technologies provide a multidimensional view of complex biological systems and have become essential tools for understanding development, homeostasis, and the cellular basis of disease.

Technological advances over the past decade have rapidly expanded the modalities, throughput, and resolution achievable in single‐cell and spatial profiling [3]. Improvements in microfluidics, barcoding chemistry, imaging, sequencing, and mass spectrometry (MS) now support deep transcriptomic, genomic, epigenomic, proteomic, and metabolomic characterization at scale [4]. Spatial platforms have progressed from multicellular spot‐level measurements to subcellular transcriptomic mapping, high‐plex protein imaging, and emerging spatial epigenomic assays [5, 6]. Joint‐profiling technologies increasingly allow simultaneous measurement of multiple molecular layers within the same cell or tissue region, enabling the reconstruction of regulatory relationships that cannot be inferred from single‐modality data alone [1, 2]. These innovations collectively generate complex, high‐dimensional datasets that require dedicated computational frameworks for accurate interpretation.

In parallel with experimental progress, computational analysis methods for single‐cell and spatial omics have expanded substantially. Classical tasks, including quality control (QC), normalization, dimensionality reduction, and clustering, remain foundational, but analytical demands now extend to tasks such as multimodal data integration, trajectory inference, gene regulatory network inference, cell–cell communication modeling, and cell niche identification [7, 8, 9, 10, 11]. Significantly, machine learning and deep learning approaches, ranging from matrix factorization and graph neural networks to variational autoencoders and transformer‐based architectures, have improved robustness to noise and enhanced cross‐modality inference [12, 13]. Moreover, recently developed foundation models trained on large‐scale cellular atlases provide unified representations across tissues and platforms, enabling transfer learning and cross‐modal prediction and offering new opportunities for biological discovery and translational research [14, 15].

These combined technological and computational developments have accelerated applications across multiple species, organ systems, and biomedical domains, spanning the development of life, oncology, immunology, neuroscience, cardiometabolic diseases, infectious diseases, and related fields, revealing disease‐associated cellular phenotypes, regulatory circuits, and microenvironmental structures inaccessible to bulk assays [16]. Such insights facilitate biomarker discovery, therapeutic target identification, and mechanistic understanding of treatment response or resistance, and are increasingly being explored in preclinical and clinical contexts [17]. Despite these advances, challenges remain in data sparsity, integration across platforms, achieving higher spatial resolution for additional modalities, and establishing standards for clinical translation.

In this review, we synthesize recent advances in single‐cell and spatial omics technologies, as well as computational and artificial intelligence (AI)‐driven analytical frameworks. We highlight their applications in human biology and disease across diverse organ systems and discuss key challenges and future directions for enhancing clinical and translational impact. The review is structured in four sections. First, we summarize breakthrough technologies for single‐cell and spatial sequencing across multiple molecular layers, including genome, epigenome, transcriptome, proteome, and metabolome. Second, we discuss computational advancements, reviewing state‐of‐the‐art methods for mono‐omics analysis, multi‐omics integration, and cross‐modal/cross‐scale data integration, with a focus on AI‐based algorithms and foundation models. Third, we summarize representative applications of these technologies in human biology and disease, illustrating how they drive biomedical discoveries. Finally, we critically discuss remaining challenges and future directions for advancing biomedical discovery and translation using single‐cell and spatial omics.

## 2 Single‐Cell and Spatial Omics: Data Modalities and Technologies

**FIGURE 1:**
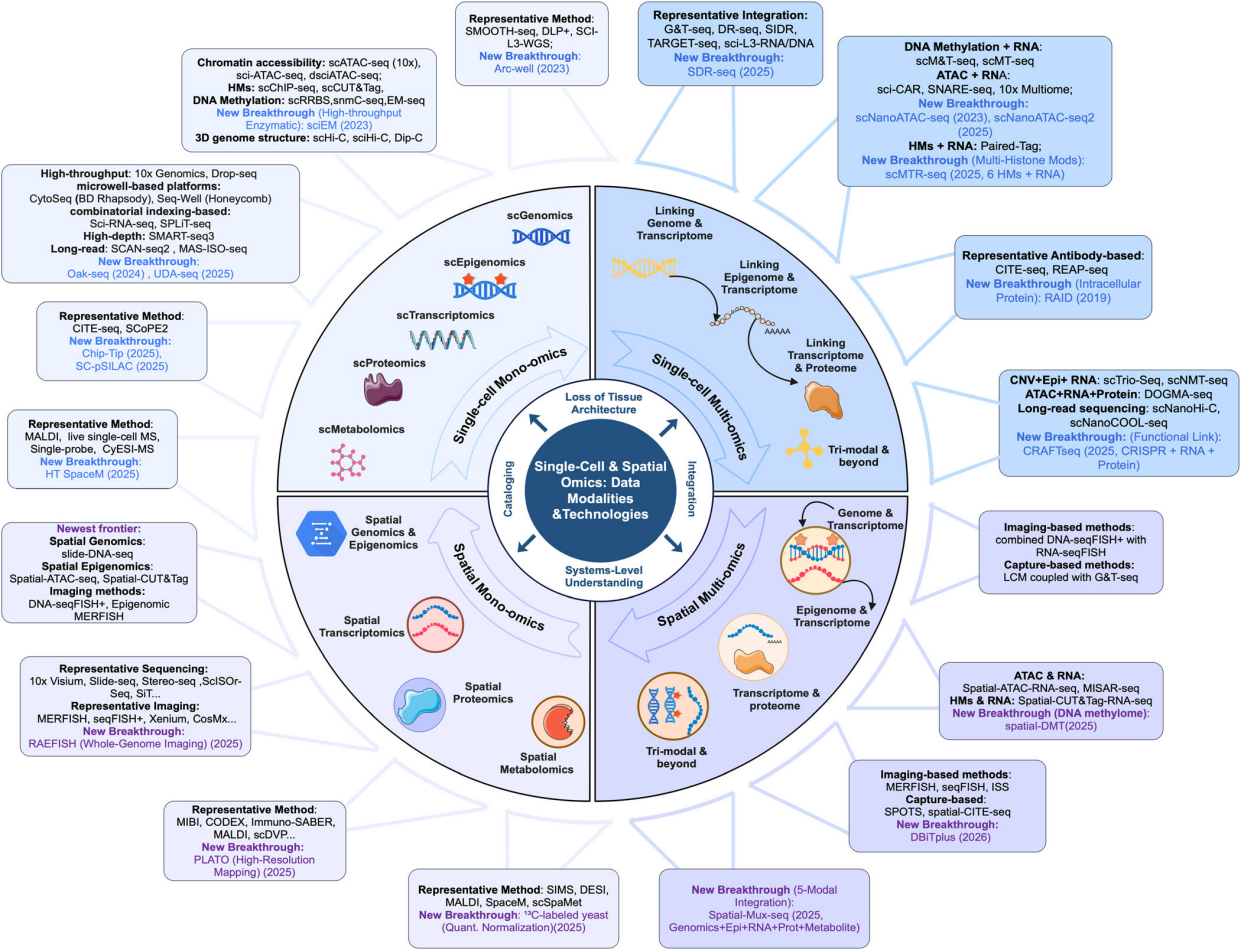
The technological landscape of single‐cell and spatial omics. This diagram illustrates the evolution of omics technologies along two primary axes: the transition from dissociated single‐cell analysis to analysis in native tissue context, and the shift from profiling single modalities to the integrative power of multi‐omics. The four quadrants categorize technologies into single‐cell mono‐omics, single‐cell multi‐omics, spatial mono‐omics, and spatial multi‐omics. The inner ring depicts the core molecular layers (genome, epigenome, transcriptome, proteome, and metabolome) and the central conceptual shift from cellular cataloging to systems‐level understanding. Outer callouts highlight both representative foundational methods and recent breakthroughs (2023–2025) designed to address critical technical trade‐offs and resolution barriers.

The architecture of multicellular organisms is inherently shaped by cellular heterogeneity. Traditional bulk‐level assays, which average molecular signals across millions of cells, obscure the very cellular diversity that governs organismal development, homeostasis, and disease. To deconstruct this complexity, a paradigm shift toward high‐resolution analysis was necessary. Single‐cell and spatial omics technologies have revolutionized our understanding of complex biological systems by enabling researchers to dissect cellular heterogeneity with unprecedented resolution. This section reviews the foundational technologies that constitute the modern single‐cell and spatial omics toolkit. These methods are the engines generating the high‐dimensional data discussed in subsequent chapters. We structure this section along two primary axes: (1) the transition from dissociated single‐cell analysis to analysis in native tissue context and (2) the evolution from profiling a single modality to the integrative power of multi‐omics (Figure 1).

### 2.1 Single‐Cell Mono‐Omic Technologies

The first technological wave aimed to resolve biological systems into their individual cellular units. This required developing methods sensitive enough to capture distinct molecular layers from vanishingly small amounts of starting material. The resulting technologies each address a fundamental biological question, but also present a unique set of technical trade‐offs.

#### 2.1.1 Single‐Cell Transcriptomics

The transcriptome serves as a comprehensive molecular profile regarding the identity and function of a cell. Single‐cell RNA sequencing (scRNA‐seq) measures genome‐wide mRNA expression in individual cells, which enables the classification of cell types, the identification of dynamic states, and the reconstruction of differentiation trajectories. This approach originated from pioneering work by Tang and colleagues [18]. Subsequently, the technological landscape of scRNA‐seq is broadly bifurcated by a crucial trade‐off between cellular throughput and transcriptomic depth. Methods that prioritize cellular scale are dominated by droplet‐based microfluidics, commercialized notably by the 10× Genomics Chromium platform [19] and building on foundational methods like Drop‐seq [20] and in Drops [21]. This approach encapsulates individual cells with barcoded beads in picoliter‐sized droplets, enabling the high‐throughput (HT) profiling of thousands to millions of cells per experiment. While cost effective and scalable, droplet‐based methods typically capture only the 3′ or 5′ end of transcripts, limiting insights into splice variants. Parallel to microfluidic droplet systems, microwell‐based platforms utilizing gravity sedimentation have achieved commercial success. The BD Rhapsody system built upon the CytoSeq method [22] and employed high‐density microwell arrays to capture cells of diverse dimensions via gravity settling. In a similar vein, the Honeycomb HIVE platform derived from Seq‐Well technology [23] and featured a portable architecture that seals the array with a semipermeable membrane. This design facilitated sample stabilization at the point of collection and thereby addressed logistical challenges in clinical studies. Furthermore, the field has expanded beyond physical compartmentalization through split‐pool combinatorial indexing (CI) strategies. Technologies commercialized by Parse Biosciences (based on SPLiT‐seq [24]) and ScaleBio (based on sci‐RNA‐seq [25]) utilized the cell or nucleus itself as a reaction vessel. By performing successive rounds of pooling and splitting to ligate barcodes, these instrument‐free methods achieved massive scalability and minimized batch effects. In contrast to these HT strategies, plate‐based methods, such as Smart‐seq2 [26] and the more recent Smart‐seq3 [27], offered higher sensitivity and full‐length transcript coverage. By processing cells in individual wells, these methods achieved superior gene detection and splicing analysis. However, this high sensitivity comes at the cost of significantly lower throughput and higher per‐cell cost, positioning these two approaches as complementary tools for different biological questions, with droplet‐seq for cellular atlasing and plate‐seq for deep, focused characterization.

However, accurately quantifying splice isoforms remains a challenge for short‐read methods. To overcome this, the field is increasingly adopting long‐read sequencing (LRS) from PacBio and Oxford Nanopore Technologies (ONT) to directly capture full‐length transcripts [28]. This strategy has been integrated into both plate‐based methods like SCAN‐seq2 [29] and droplet‐based adaptations like MAS‐ISO‐seq [30], substantially enabling the isoform complexity that standard protocols miss. Parallel to these gains in resolution, other breakthroughs seek to multiply throughput by creating hybrid strategies. Technologies like OAK‐seq [31] and UDA‐seq [32] employed a combinatorial droplet‐based barcoding approach. They innovatively combined a 10× droplet‐barcoding step with a secondary plate‐barcoding step. This two‐step combinatorial method dramatically increased cell throughput per channel (e.g., >150,000 cells) while maintaining high data quality and multi‐omics compatibility, further pushing the boundaries of scale.

#### 2.1.2 Single‐Cell Genomics

Single‐cell DNA sequencing (scDNA‐seq) interrogates the genome of individual cells to resolve clonal architecture and somatic heterogeneity, including copy number variations (CNVs) and mutation patterns that are neglected in bulk analyses. Given the picogram‐scale DNA content (∼6 pg) within a single cell, whole‐genome amplification (WGA) is a requisite step. However, WGA represents the principal bottleneck of this technology due to technical artifacts like amplification bias and allele dropout.

Strategies for WGA exhibited inherent technical trade‐offs. Isothermal amplification methods, such as multiple displacement amplification (MDA) [33], provided higher genome coverage but exhibited uneven amplification that impeded the accurate calling of CNVs. Conversely, PCR‐based methods exemplified by DOP‐PCR [34] and hybrids like multiple annealing and looping‐based amplification cycles (MALBAC) [35, 36] offered improved uniformity but suffered from reduced coverage. Contemporary innovations in WGA aimed to mitigate these limitations. For instance, PTA [37] improved the uniformity associated with MDA, while META‐CS [38] enhanced the accuracy for SNV detection through the sequencing of complementary DNA strands.

Reconstructing tumor phylogeny requires HT platforms. Methodologies have evolved beyond low‐throughput methods by integrating CI with linear amplification [39], as seen in sci‐L3‐WGS, which built upon Linear Amplification via Transposon Insertion [40]. Alternative approaches circumvented WGA entirely by using direct tagmentation within microfluidic or nanowell platforms like DLP+ [41]. Notably, recent HT platforms like Arc‐well [42] facilitated scWGS on archival FFPE tissues, thereby overcoming a major translational barrier. These advancements transitioned scDNA‐seq from a basic research tool into a robust modality for tracking clonal evolution in clinical samples. Nevertheless, the resolution of complex structural variants (SVs) and long‐range haplotype information remained a major hurdle for short‐read platforms. To address this limitation, recent strategies have integrated scWGA with LRS. Pioneering methods like SMOOTH‐seq [43] first combined scWGS with PacBio sequencing to profile SVs. Subsequent advancements included droplet‐based PacBio approaches for identifying somatic mutations [44] and ONT‐based methods for chromosome‐wide haplotype phasing [45], revealing a layer of genomic variation missed by short‐read platforms.

#### 2.1.3 Single‐Cell Epigenomics

The epigenome regulates cell‐type‐specific gene expression through diverse features, including chromatin accessibility, histone modifications (HMs), and DNA methylation. Profiling these at single‐cell resolution is crucial for understanding regulatory mechanisms but faces fundamentally challenges regarding extreme data sparsity.

The most mature technology in this domain is scATAC‐seq, which profiles chromatin accessibility using Tn5 transposase [46]. The evolution of this method was driven by throughput, rapidly scaling from early microfluidic methods [47] to HT CI approaches such as sci‐ATAC‐seq [48] and droplet‐based CI methods including dsciATAC‐seq [49]. Profiling HMs and TF binding presents greater challenges. Early scChIP‐seq methods demonstrated low efficiency [50]. Consequently, the field shifted toward sensitive, low‐input methods based on CUT&RUN [51], leading to the widely adopted scCUT&Tag [52]. Significantly, the high sensitivity of Tn5‐based tagmentation in scCUT&Tag enabled profiling of HMs and precise mapping of genomic binding sites for abundant TFs, such as Pol II, which was challenging with previous methods. For DNA methylation, bisulfite‐based methods such as scRRBS [53] and the HT snmC‐seq [54] remained the gold standard. However, these methods induced DNA damage. Newer enzymatic conversion methods have emerged as a key alternative. Approaches such as EM‐seq [55] and the recent HT sciEM [56] utilized enzymes to convert unmethylated cytosines, thereby preserving DNA integrity and offering a robust alternative to bisulfite.

Beyond these linear epigenetic marks, gene regulation is fundamentally governed by higher‐order chromatin structure. To resolve the three‐dimensional (3D) genome, single‐cell Hi‐C (scHi‐C) [57] and HT variants such as sciHi‐C [58] and Dip‐C [59] were developed. These methods captured genome‐wide chromatin interactions and revealed cell‐to‐cell heterogeneity in 3D architectural features, including topologically associating domains and enhancer‐promoter looping, providing another critical dimension of epigenetic control.

#### 2.1.4 Single‐Cell Proteomics

Profiling proteins, the functional effectors of the cell, presents unique challenges as these molecules cannot be amplified. This fundamental limitation, coupled with the low starting amounts (∼100–200 pg) [60], has directed the field toward two distinct methodological strategies. The first strategy involved target‐based methods, which relied on antibodies for specificity. This included antibody‐based assays like CyTOF [61] and PCR‐sequencing based assays, represented by  CITE‐seq [62]. While these hypothesis‐driven methods offered high throughput and seamless integration, they remained limited by the availability of specific antibodies. The second strategy utilized unbiased, discovery‐driven MS. Historically, this approach encountered significant challenges regarding sensitivity and throughput. Although early breakthroughs such as SCoPE‐MS [63] and SCoPE2 [64] established the foundation, the field advanced into an era of deep proteomics in 2025. This progression was driven by new instrumentation, such as the Orbitrap Astral mass spectrometer, and was illustrated by two concurrent studies: Bubis et al. [65] identified over 5300 proteins in single A549 cells, while the Chip‐Tip workflow [66] detected over 5000 proteins in HeLa cells, quantifying approximately half the expressed proteome.

Moreover, previous methods provided only a static snapshot of protein abundance. A distinct breakthrough in 2025, SC‐pSILAC [67], addressed this limitation by adapting pulsed‐SILAC for single‐cell scale, enabling simultaneous quantification of protein abundance and turnover dynamics (L/H ratio) and measuring approximately 3000 proteins. Collectively, these advances are rapidly driving MS‐based methods toward greater depth and functional insight.

#### 2.1.5 Single‐Cell Metabolomics

Metabolites represent the final functional output of a cell's state; however, profiling this modality remains exceptionally challenging. The metabolome is characterized by extreme chemical diversity, low abundance, rapid turnover in milliseconds, and cannot be amplified [68]. Consequently, MS serves as the dominant tool, as alternative microscopy‐based methods lacked biochemical information and spectroscopy techniques such as NMR lacked sensitivity [69].

Within MS‐based single‐cell metabolomics (SCM), the core challenge involves the sampling strategy, which was broadly divided into three categories [70]. Desorption Ionization, such as MALDI, acted in situ and offered high throughput, but exhibited low coverage of small molecules [71]. Content extraction, including live single‐cell MS [72] and the single‐probe [73], offered high sensitivity but demonstrated extremely low throughput [74]. Sorting and ionization approaches, exemplified by CyESI‐MS [75], aimed for high throughput but involved complex cell handling. A recent breakthrough, HT SpaceM [76], directly addressed the limitations of this in situ strategy. By advancing MS imaging technology, HT SpaceM provided a HT platform (>140,000 cells per slide) with high coverage of small molecules and high reproducibility. This technology transitioned SCM from niche exploration to a tool capable of large‐scale analysis of clinical samples.

### 2.2 Joint Profiling of Single‐Cell Multi‐omics

Profiling a single modality, as reviewed in Section 2.1, provides only a limited perspective. However, the cellular state emerges from the complex interplay among the genome, epigenome, transcriptome, and proteome. Consequently, a primary objective within the field involves the simultaneous measurement of these modalities from the same cell. This approach aims to transition from correlation to causality by directly linking regulatory mechanisms, such as connecting an enhancer to a target gene, thereby providing a more robust definition of cellular identity. This section reviews key single‐cell multi‐omic strategies and focuses on technical solutions for connecting these distinct molecular layers.

#### 2.2.1 Linking the Genome and Transcriptome

A central objective within multio‐mics involves the direct bridging of the genotype with the transcriptional phenotype. This integration remains essential not only for resolving somatic heterogeneity, such as the investigation of oncogenic drivers in cancer, but also for elucidating germline genetics. Connecting inherited variants, specifically single nucleotide polymorphisms (SNPs), to single‐cell expression profiles facilitates the mapping of expression quantitative trait loci (eQTLs) and the assessment of allelic effects with resolution specific to cell types [77, 78, 79]. Such analyses enable researchers to dissect how specific genetic variants influence gene regulation in a context‐dependent manner.

The primary technical barrier involves the physical separation or distinct processing of DNA and RNA from the identical cell prior to analysis. Foundational, plate‐based methods addressed this challenge through various isolation strategies. For example, G&T‐seq [80] utilized oligo‐dT beads to capture the poly(A) mRNA, leaving the genomic DNA behind in the lysate for separate processing. Similarly, SIDR [81] employed hypotonic lysis to physically separate the nucleus containing DNA from the cytoplasm containing RNA. Alternative approaches, such as DR‐seq [82], involved the amplification of nucleic acids prior to splitting the sample for parallel sequencing. Building upon these foundations, TARGET‐seq [83] optimized the isolation and reverse transcription steps, which achieved higher sensitivity for mutation detection and improved throughput relative to earlier protocols. While these methods successfully solved the separation issue, they remained limited in cellular throughput. To overcome this constraint, the field adopted HT CI. A prominent example is the sci‐L3‐RNA/DNA coassay, which profiled tens of thousands to over a million nuclei [39]. However, this massive scale often comes at the cost of per‐cell sensitivity. Recent advances, such as the droplet‐based SDR‐seq method [84], addressed this limitation by minimizing allelic dropout. This method demonstrated the ability to achieve reliable zygosity calls from the majority of cells, as well as the interrogation of non‐coding variants for both somatic and germline analysis.

#### 2.2.2 Linking the Epigenome and Transcriptome

This integration represents the most established multi‐omic modality and aims to elucidate how regulatory elements modulate gene expression. Methodologies are categorized based on the specific epigenetic features targeted. Early plate‐based methods, such as scM&T‐seq [85] and scMT‐seq [86], focused on DNA methylation. These protocols achieved dual profiling by physically separating genomic DNA for bisulfite sequencing from mRNA; however, reliance on plates constrained throughput. Consequently, the field rapidly shifted toward chromatin accessibility, the primary modality for linking cis‐regulatory elements to target genes. Technologies evolved from the initial sci‐CAR method [87] to approaches such as SNARE‐seq [88], which enhanced the sequencing coverage, and SHARE‐seq [89], which improved sensitivity and scalability. This progression culminated in the commercial 10x Genomics Multiome platform (https://www.10xgenomics.com/cn/blog/introducing‐chromium‐single‐cell‐multiome‐atac‐gene‐expression). Coupling HMs with the transcriptome was more complex. Foundational methods such as Paired‐Tag [90] established the proof‐of‐concept; however, simultaneous profiling of multiple marks remained a significant technical challenge. A breakthrough in 2025, scMTR‐seq, addressed this limitation by simultaneously capturing six distinct HMs and the full transcriptome [91]. The high sensitivity and cell recovery rate of this method provided a powerful new tool for dissecting complex epigenetic regulation.

While these methods link epigenomic features to transcription, a parallel challenge involves connecting the epigenome directly to underlying genomic variants, such as SVs, which are often missed by short‐reads sequencing. LRS now bridges this gap. For instance, scNanoATAC‐seq [92] leveraged LRS to jointly profile chromatin accessibility and genetic variants from the same read. This LRS approach also addressed critical challenges regarding sample sensitivity. The recent scNanoATAC‐seq2 [93] offered a solution for scarce samples by utilizing an innovative one‐pot reaction to yield high‐quality, multi‐omic accessibility and genotype data from single cells.

#### 2.2.3 Linking the Transcriptome and Proteome

A well‐established challenge in biology is that mRNA abundance often serves as a poor surrogate for protein level. This multi‐omic integration directly addresses that discrepancy. The field is characterized by methods leveraging oligonucleotide‐labeled antibodies as a surrogate signal. CITE‐seq [62] and REAP‐seq [94] standardized this approach by co‐capturing antibody tags alongside mRNA within a standard scRNA‐seq workflow, such as the 10× Genomics platform. This strategy provided a robust definition of cellular identity by combining protein markers with the full transcriptome. A primary limitation involved the reliance on antibody availability and a focus on cell surface proteins. This limitation was addressed by methods such as RAID [95], which extended the concept to more challenging intracellular proteins.

#### 2.2.4 Higher‐Order Integration

The technical frontier lies in tri‐modal or higher‐order profiling, which seeks to capture a systems‐level view of a single cell. The core challenge involves integrating these disparate molecular layers, a process that often amplifies data sparsity. Foundational methods combined three layers, frequently focusing on DNA and RNA. For example, scTrio‐seq [96] simultaneously measured the genome (CNV), DNA methylome, and transcriptome. Similarly, scNMT‐seq captured the transcriptome, DNA methylome, and chromatin accessibility [97]. More recent methodologies have successfully integrated proteomics. DOGMA‐seq [98], for instance, linked chromatin accessibility (ATAC), RNA, and proteins. Similarly, the CRAFT‐seq platform [99] integrated transcriptome and surface protein profiling with the direct detection of CRISPR gene editing outcomes, thereby functionally linking genotype to phenotype within a single cell.

The capabilities of LRS have also begun to enhance these higher‐order integration strategies. LRS platforms facilitate the resolution of complex genomic features alongside other modalities. For example, scNanoHi‐C [100] leverages LRS to profile 3D chromatin contacts and genomic variants (multimodal epigenomic/genomic insights) simultaneously. This trend toward LRS‐based higher‐order integration is best exemplified by platforms such as scNanoCOOL‐seq [101]. This method profiled four modalities in the same cell, including CNVs, DNA methylome, and chromatin accessibility via LRS, alongside the transcriptome via short‐reads. This approach realized the goal of a comprehensive, high‐resolution cellular profile. These complex methods highlight the trajectory of the field toward increasingly integrated molecular characterizations.

In summary, the technologies reviewed thus far mark a major conceptual leap. Mono‐omic methods provided essential tools to catalog isolated molecular layers and classify cellular states. Multi‐omic methods subsequently delivered the power to connect these layers. This crucial evolution from classification to integration enables the field to move beyond cataloging composition toward elucidating regulatory mechanisms, providing a true systems‐level view of cellular identity.

### 2.3 Spatial Omics: Data Modalities and Technologies

The analysis of dissociated cells, while revolutionary for dissecting heterogeneity, incurs the fundamental cost of losing all information regarding tissue architecture. Cellular function is dictated not only by internal state but also profoundly by spatial positioning and the cellular neighborhood. To address this limitation, spatial omics technologies emerged to capture molecular information in situ. This section reviews the core technologies for profiling distinct modalities within their native context, facilitating a paradigm shift from characterizing cellular identity to mapping spatial localization.

#### 2.3.1 Spatial Transcriptomics

Spatial transcriptomics (ST) represents the most mature spatial modality; however, the field is characterized by a fundamental trade‐off between resolution and plexity (throughput and coverage). The first major strategy, sequencing‐based or capture‐based methods, aimed for unbiased, whole‐transcriptome coverage. This trajectory evolved significantly regarding barcode carrier technology to address resolution limitations. Early methods, like ST [102] and 10× Visium, utilized spatially barcoded arrays but were limited to multicellular spots. To address this resolution constraint, subsequent approaches increased barcoding density using higher‐density substrates, such as bead‐based technologies of Slide‐seq [103] and HDST [104]. This evolution culminated in nanoball‐array technologies, exemplified by Stereo‐seq [105], which offered subcellular resolution. Nevertheless, a limitation of these sequencing‐based approaches was that high resolution led to reduced capture sensitivity in terms of UMIs per cell. The second critical limitation involved the reliance on short‐reads, which failed to resolve spatial isoform diversity. To address this gap, recent breakthroughs bridged spatial barcoding with LRS. Methods such as ScISOr‐Seq [106] and the more recent SiT [107] facilitated the creation of spatially resolved splicing maps, linking specific isoforms to distinct tissue domains.

In contrast, imaging‐based or in situ methods prioritized high sensitivity and high resolution. These approaches built upon single‐molecule FISH (smFISH) [108], which offered exceptional sensitivity but was constrained by extremely low multiplexing capacity. The central challenge of scaling this method to thousands of genes was overcome through multiplexing, utilizing either combinatorial barcoding, exemplified by MERFISH [109], or sequential hybridization, as used in seqFISH/seqFISH+ [110]. These technologies have been successfully commercialized into platforms such as Xenium and CosMx [111].

This dichotomy established a fundamental challenge: achieving genome coverage at lower resolution via sequencing versus high resolution for targeted genes via imaging. A recent advancement, RAEFISH (Reverse‐padlock Amplicon Encoding Fluorescence In Situ Hybridization), directly resolved this trade‐off. RAEFISH is a sequencing‐free, imaging‐based method that achieved both whole‐genome coverage, detecting over 22,000 genes in mouse models, and single‐molecule resolution. This capability provided an unbiased, high‐resolution view of the transcriptome in situ [112].

#### 2.3.2 Spatial Proteomics

While single‐cell proteomics encounters the inherent challenge of non‐amplification, spatial proteomics presents the additional challenge of achieving high multiplexing capacity in situ [113]. The dominant approach relied on antibody‐based targeted strategies, which evolved from traditional immunofluorescence (IF) and its limitation in detecting only three to five proteins. To address this spectral limitation, strategies expanded beyond fluorescence. Multiplexed ion beam imaging (MIBI) [114] utilized heavy metal isotopes for approximately 40‐plex imaging, whereas scalable DNA‐barcoded antibody methods, such as CODEX [115] and Immuno‐SABER [116], employed sequential hybridization to visualize hundreds of proteins [117]. A primary limitation of these approaches stemmed from their reliance on antibody‐mediated targeting, which constrains both the number of proteins amenable to detection and the spatial resolution of the assay. For unbiased, discovery‐driven proteomics, mass spectrometry imaging (MSI), such as MALDI [118] was employed, which involved a trade‐off regarding low spatial resolution. However, advances such as scDVP [119] integrated laser microdissection with MS to advance this discovery approach toward single‐cell resolution. Recently, novel platforms such as PLATO further advanced high‐resolution mapping by combining microfluidics with deep learning algorithms to analyze complex tumor microenvironments [120].

#### 2.3.3 Spatial Metabolomics

The inherent challenges of SCM are compounded in spatial analysis by the requirement for in situ identification. The field relied on unbiased MSI [121], using various ionization methods including SIMS [122], DESI [123], and MALDI [118]. However, these MSI methods historically encountered two primary bottlenecks: low spatial resolution and poor quantitation attributable to matrix and batch effects. Progress toward improved resolution was achieved through pioneering computational frameworks. SpaceM [124] integrated MSI data with high‐resolution fluorescence and morphological images, employing computational segmentation to assign metabolic signals to single cells. The scSpaMet framework [125] further advanced this approach by integrating multiplexed protein profiling, which enabled the assignment of metabolites to specific cell types. Recently, the quantitation limitation was alleviated by a novel normalization strategy utilizing a ^1^ ^3^C‐labeled yeast extract as a universal internal standard, thereby facilitating robust comparative and quantitative analysis across samples [126].

#### 2.3.4 Spatial Genomics and Epigenomics

Profiling the genome and epigenome in situ represents an emerging frontier. The core challenge involved the fact that analytical methods for genomics, such as WGA, and epigenomics, such as Tn5 tagmentation, typically necessitated cell lysis or harsh chemical conditions that are fundamentally incompatible with the preservation of tissue integrity. Current strategies adapted established single‐cell protocols to spatial platforms. For spatial genomics, aimed at mapping clonal CNVs, slide‐DNA‐seq [127] adapted the Slide‐seq bead platform to capture gDNA. Regarding spatial epigenomics, technologies including Spatial‐ATAC‐seq [128] and Spatial‐CUT&Tag [129] were developed. These methods coupled in situ enzymatic reactions (Tn5 transposition or antibody‐tethered pA‐Tn5) with spatial barcoding, often via microfluidics as exemplified by DBiT‐seq [130], to capture the chromatin state. As an alternative to sequencing, imaging methods such as DNA‐seqFISH+ [131] and epigenomic MERFISH [132] utilized probe hybridization to visualize the 3D organization of chromatin loci directly.

### 2.4 Joint Profiling of Spatial Multi‐omics

While mono‐omic spatial methods provide critical maps, they offer isolated views of cellular function. Understanding the in situ mechanisms of the Central Dogma, specifically how genomic regulation translates to functional protein output, necessitates the simultaneous capture of multiple molecular layers. This nascent field is rapidly evolving to address this challenge, progressing from dual‐modal pairings to higher‐order systems integration.

In alignment with the flow of genetic information, the first key axis links the genome and epigenome to the transcriptome. Imaging‐based methods combined DNA‐seqFISH+ with RNA‐seqFISH and antibody staining (IF) to visualize the genome, transcriptome, and proteins within the same nucleus [131]. Capture‐based methods, such as laser capture microdissection coupled with G&T‐seq [133], can also retrieve genomic and transcriptomic data from defined regions. However, the most active area involves linking epigenetic state to gene expression. Technologies such as Spatial‐ATAC‐RNA‐seq and Spatial‐CUT&Tag‐RNA‐seq [129, 134] were developed to profile chromatin accessibility and HMs alongside RNA, which leveraged microfluidic barcoding platforms as exemplified by DBiT‐seq [130]. The MISAR‐seq [135] platform advanced this by utilizing microfluidic indexing to coprofile ATAC and RNA in the developing mouse brain. A significant advancement, spatial‐DMT [136], enabled access to the critical layer of the DNA methylome, achieving the first joint spatial profiling of whole‐genome DNA methylation and the transcriptome within the same tissue slide.

The second major axis connects the transcriptome to the proteome, thereby linking genetic blueprints to functional effectors. This integration has also evolved along two distinct paths. Imaging‐based methods combined high‐resolution in situ RNA detection, such as MERFISH, seqFISH, or ISS, with multiplexed IF [109, 131, 137]. Separately, capture‐based platforms were adapted for dual‐omics. This originated with simple overlays of low‐plex IF on 10x Visium and evolved to higher‐plex methods. Platforms such as NanoString GeoMx DSP (NanoString: https://nanostring.com/products/geomx‐digital‐spatial‐profiler/geomx‐dsp‐overview/) and SM‐Omics [138] enabled high‐plex digital protein readouts alongside RNA. The integration of antibody‐derived oligonucleotide tags (ADTs) further advanced this field, enabling methods such as SPOTS [139] and spatial‐CITE‐seq [140] to capture proteins and RNA within a unified sequencing workflow. Recent development of DBiTplus combined sequencing‐based ST and multiplexed protein imaging on the same section, which enabled unbiased analysis of whole‐transcriptome and proteome at the spatial level [141].

The technical frontier is currently moving beyond dual‐omics toward higher‐order, systems‐level integration. Early examples combined high‐resolution imaging to visualize the genome, transcriptome, and nuclear proteins simultaneously [142]. A significant advancement represented the recent development of Spatial‐Mux‐seq [143], which transcended the dual‐omic limitation and simultaneously mapped five distinct modalities, involving chromatin accessibility, two HMs, the whole transcriptome, and a panel of proteins within the same tissue section.

**FIGURE 2:**
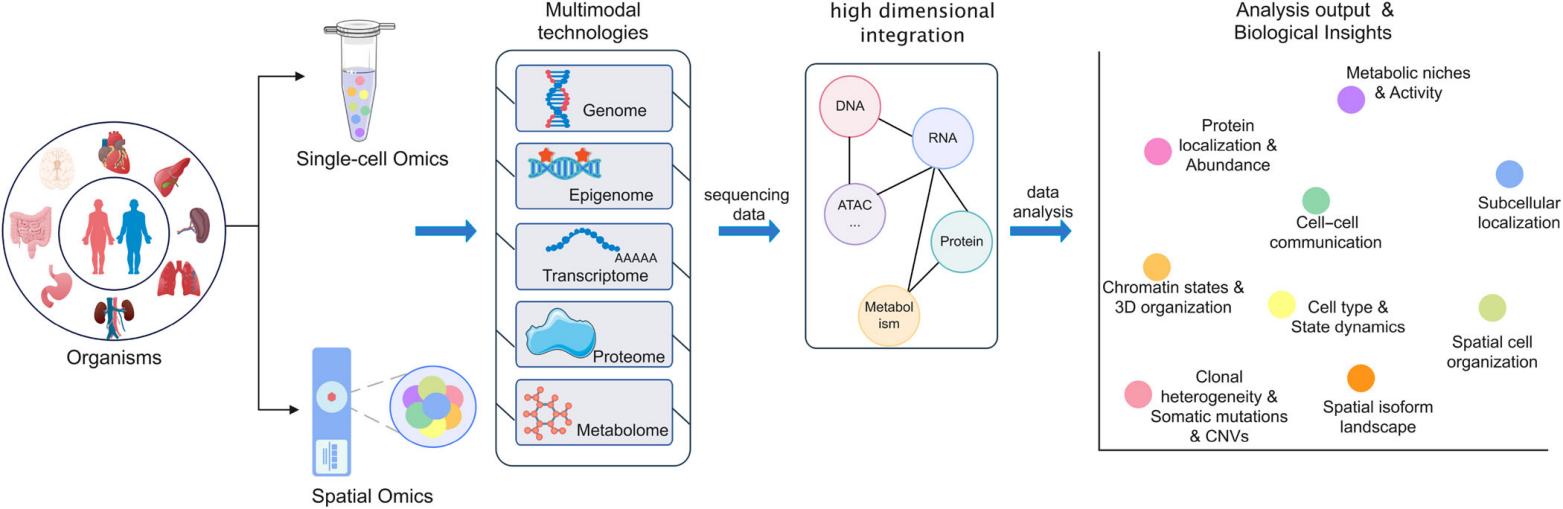
Workflow from biological samples to biological insights in single‐cell and spatial omics. This diagram illustrates the comprehensive pipeline of single‐cell and spatial omics, starting from biological samples (dissociated cells or intact tissue). Multimodal technologies are applied to profile various molecular layers (genome, epigenome, transcriptome, proteome, and metabolome), generating high‐dimensional data. These data undergo sophisticated computational integration to derive key biological insights and analysis outputs, including clonal heterogeneity, cell identity, spatial architecture, and functional dynamics within complex biological systems.

The rapid evolution of these technologies, particularly for co‐profiling the epigenome and transcriptome such as spatial‐DMT and Spatial‐Mux‐seq, is generating datasets of unprecedented complexity and scale. These massive, multimodal spatial data pose a significant challenge, necessitating the development of novel computational and AI‐driven strategies, which will be discussed in Section 3, for their integration and biological interpretation (Figure 2).

## 3 Single‐Cell and Spatial Multiomics Analysis: Algorithms, AI Methods, and Foundation Models

**FIGURE 3:**
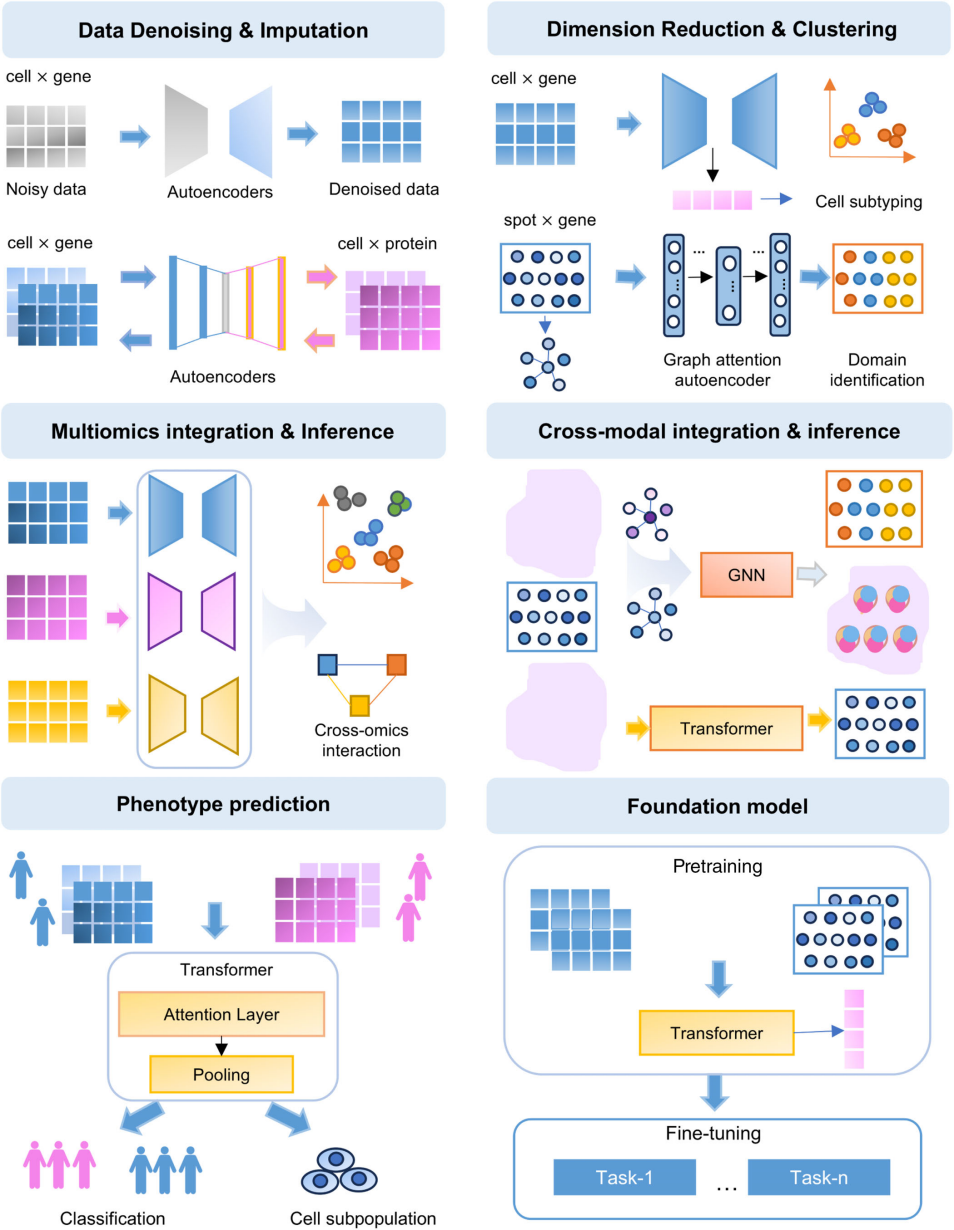
AI‐based algorithms for single‐cell and spatial omics analysis. Existing AI‐based algorithms can be classified into six types according to their function. (1) Data denoising and imputation: some algorithms performed data denoising directly on the single‐cell omics data, while others performed imputation from other types of single‐cell omics data. (2) Dimension reduction and clustering: autoencoder also facilitated the cell type clustering or spatial domain identification by learning latent representation from single‐cell or spatial omics data. (3) Multi‐omics integration and Inference: multimodal autoencoders enabled multi‐omics integration and identified cross‐modal interaction. (4) Cross‐modal integration and inference: integrated spatial omics with histopathology image using graph‐based neural network improved spatial domain identification. Transformer provided cross‐modal inference from histopathology images to spatial omics. (5) Phenotype prediction: transformer linked single‐cell omics with clinical phenotypes and facilitated the phenotype‐specific subpopulation identification. (6) Foundation model: developing foundation models on large‐scale single‐cell or spatial omics data facilitated multiple downstream tasks by pretraining and fine‐tuning paradigm.

The generation of massive single‐cell and spatial multi‐omics data necessitates the development of a variety of algorithms to tackle the sparsity and high dimensional issues of data. Compared with traditional methods that rely on experience and parameter tuning, AI especially deep learning suggests powerful methods in dealing with these data issues, providing efficient tools for data imputation, dimension reduction, and clustering [12]. AI can also overcome the heterogeneity among different layers of data, facilitating the effective integration of multi‐omics, cross‐modal data, and linking cell‐level or spatial‐level omics data with patient‐level phenotypes. Recent foundation modeling techniques and agents further extended the power of AI, facilitating the analysis of multiple tasks in an effective, efficient and automated way [144]. These recent breakthroughs in single‐cell and spatial omics analysis have been summarized in the following subsections, along with Figure 3 and Tables 1 and 2.

**TABLE 1:** Algorithms for single‐cell and spatial omics analysis.

Algorithm/model	Analysis type	Omics type	Architecture/method	Year	Application/function
HiScanner [145]	Single‐cell genome	scWGS	High‐resolution scanning	2025	Detection of somatic copy number alterations (CNAs) from low‐coverage scWGS
SCICoNE [146]	Single‐cell genome	scDNA‐seq	Statistical model + MCMC	2025	Reconstruction of copy number event history from shallow sequencing data
MEDALT [147]	Single‐cell genome	scDNA‐seq	Lineage tracing	2021	Discovery of fitness‐associated alterations via single‐cell lineage construction
Numbat [148]	Single‐cell genome	scRNA‐seq	Haplotype‐aware phasing	2023	Inference of CNVs from transcriptomic data
SComatic [150]	Single‐cell genome	scRNA‐seq, scATAC‐seq	Statistical model	2024	Detection of SNPs from single‐cell RNA‐seq data
Cellsnp‐lite [151]	Single‐cell genome	scRNA‐seq, scATAC‐seq	Statistical model	2021	Detection of SNPs from single‐cell ATAC‐seq data
CopyKAT [152]	Single‐cell genome	scRNA‐seq	Bayesian segmentation (statistical)	2021	CNV inference using only expression matrix input
CopyVAE [154]	Single‐cell genome	scRNA‐seq	VAE	2024	Deep learning‐based inference of CNVs
SmartImpute [158]	Single‐cell transcriptome	scRNA‐seq	targeted imputation framework	2025	Perform imputation on missing data
scVGAMF [159]	Single‐cell transcriptome	scRNA‐seq	variational graph autoencoder, non‐negative matrix factorization	2025	Perform imputation on missing data
DCA [160]	Single‐cell transcriptome	scRNA‐seq	Deep count autoencoder	2019	Data denoising
GraphSCI [161]	Single‐cell transcriptome	scRNA‐seq	GCN + autoencoder	2021	Perform imputation on missing data
SAVER‐X [162]	Single‐cell transcriptome	scRNA‐seq	Transfer learning	2019	Data denoising
scVI [164]	Single‐cell transcriptome	scRNA‐seq	VAE	2018	Dimension reduction and clustering
Scvis [166]	Single‐cell transcriptome	scRNA‐seq	Deep generative model/statistical model	2018	Dimension reduction and clustering
scDeepCluster [168]	Single‐cell transcriptome	scRNA‐seq	Autoencoder	2019	Dimension reduction and clustering
d‐scIGM [170]	Single‐cell transcriptome	scRNA‐seq	Deep interpretable generative model	2024	Dimension reduction and clustering
BERMUDA [172]	Single‐cell transcriptome	scRNA‐seq	Autoencoder	2019	Batch correction and annotation
MARS [174]	Single‐cell transcriptome	scRNA‐seq	Deep learning (meta‐learning)	2020	Annotation
scArches [175]	Single‐cell transcriptome	scRNA‐seq	Transfer learning	2022	Mapping
mtSC [176]	Single‐cell transcriptome	scRNA‐seq	Multitask deep metric learning	2021	Single‐cell assignment
VITAE [180]	Single‐cell transcriptome	scRNA‐seq	VAE + latent mixture model	2024	Trajectory inference
PRESCIENT [181]	Single‐cell transcriptome	scRNA‐seq	Deep generative model	2021	Trajectory inference
CellOT [182]	Single‐cell transcriptome	scRNA‐seq	Optimal transport + convex neural networks	2023	Perturbation
Scissor [183]	Single‐cell transcriptome	Bulk + scRNA	Regression	2022	Phenotype association
PENCIL [184]	Single‐cell transcriptome	scRNA‐seq	Learning with rejection (LWR)	2023	Phenotype association
ScRAT [185]	Single‐cell transcriptome	scRNA‐seq	Attention‐based neural networks	2024	Phenotype association
Quantum annealing methods [187]	Single‐cell transcriptome	scRNA‐seq	Quantum annealing	2023	Data clustering
EpiScanpy [193]	Single‐cell epigenome	scATAC/single‐cell DNA methylation	K‐nearest neighbor (KNN) graph	2021	Single‐cell epigenomic analysis toolkit
SnapATAC [194]/SnapATAC2 [195]	Single‐cell epigenome	scATAC‐seq	Scalable framework/spectral embedding	2021/2024	Single‐cell epigenomic analysis toolkit
scDEC‐Hi‐C [199]	Single‐cell epigenome/3D genome	scHi‐C	Convolutional autoencoder (CAE)	2023	Imputation and deep embedding clustering of scHi‐C data
Higashi [200]	Single‐cell epigenome/3D genome	scHi‐C	Hypergraph representation learning	2022	Imputation and embedding of scHi‐C data by capturing latent structures in 2D contact maps
scHiMe [201]	Single‐cell epigenome	scHi‐C/single‐cell DNA methylation	Graph transformer	2023	Single‐cell DNA methylation prediction
ConvNet‐VAEs [202]	Single‐cell epigenome	Single‐cell multimodal epigenomic datasets	1D CNN + VAE	2024	Integration of histone modification and chromatin accessibility data
scPROTEIN [204]	Single‐cell protein	Single‐cell proteomics	Deep contrastive learning	2024	Proteomics embedding
PINNACLE [205]	Single‐cell protein	Single‐cell proteomics	Geometric deep learning	2024	Protein interaction
BABEL [207]	Single‐cell protein	scRNA/ATAC/Proteomics	Multiple autoencoders	2021	Cross‐modality translation
SCMeTA [206]	Single‐cell metabolomics	Single‐cell metabolomics	Statistical model	2024	Preprocessing and analytical pipelines
GEFMAP [209]	Single‐cell metabolomics	Single‐cell metabolomics	Geometric deep learning	2024	Predict metabolic states from gene expression
CalicoST [210]	Spatial genome	SRT	Statistical inference	2024	Inference of allele‐specific CNAs and reconstruction of spatial tumor evolution
SlideCNA [211]	Spatial genome	Slide‐seq	Expression‐aware binning	2025	Recovery of CNA patterns from sparse spatial transcriptomics to detect subclonal regions
spaPeakVAE [213]	Spatial epigenome	Spatial ATAC	VAE	2024	Capture spatial correlations among spots to improve spatial ATAC analysis
SpaGCN [217]	Spatial transcriptome	SRT	Graph convolutional network (GCN)	2021	Integration of gene expression, histology, and location to identify domains and spatially variable genes
BayesSpace [215]	Spatial transcriptome	SRT	Bayesian statistical model	2021	Resolution enhancement and clustering utilizing spatial neighborhood information
stLearn [216]	Spatial transcriptome	SRT + image	Graph‐based framework	2023	Imputation and infers spatial trajectory
STAGATE [218]	Spatial transcriptome	SRT	Graph attention auto‐encoder	2022	Identification of spatial domains via learning latent embeddings from spatial and expression data
GraphST [219]	Spatial transcriptome	SRT	Graph self‐supervised contrastive learning	2023	Spatial clustering and correcting batch effects
SPARK‐X [221]	Spatial transcriptome	SRT	Nonparametric framework	2025	Detection of spatially variable genes (SVGs) in large‐scale datasets
SCS [223]	Spatial transcriptome	SRT + image	Transformer	2023	High‐resolution cell segmentation combining imaging and sequencing data
Bering [224]	Spatial transcriptome	SRT	Graph deep learning	2025	Segmentation and annotation
UCS [225]	Spatial transcriptome	SRT	Convolutional neural networks	2025	Cell segmentation for subcellular spatial transcriptome
CellCharter [226]	Spatial transcriptome	SRT	VAE/Gaussian mixture model (GMM)	2024	Identification and characterization of spatial cellular niches and tissue remodeling
scNiche [227]	Spatial transcriptome	SRT	Multiple graph autoencoder (M‐GAE)	2025	Identification and characterization of cell niches in tissue
NicheCompass [228]	Spatial transcriptome	SRT	Graph neural network (GNN)	2025	Learning interpretable cell embeddings to query spatial niches
AnnoSpat [234].	Spatial proteomics	Spatial proteomics	Extreme learning machine (ELM) classifier	2024	Annotation and quantify cell–cell proximity relationships
STARLING [235]	Spatial proteomics	Spatial proteomics	Probabilistic machine learning model	2025	Quantifying cell populations and phenotypes
SmartGate [238]	Spatial metabolomics	Spatial metabolomics	Graph attention autoencoder	2023	Automatic peak selection and spatial structure identification
MOFA+ [240]	Integration	Single‐cell multiomics	Matrix factorization	2020	Projection of multiomics data into a shared low‐dimensional latent space
UnpairReg [241]	Integration	Unpaired omics	Regression analysis	2022	Estimation of scRNA‐seq from scATAC‐seq using biological association constraints
GLUE [242]	Integration	Unpaired omics	Graph‐linked embedding + adversarial learning	2022	Integration of unpaired multiomics data incorporating regulatory knowledge graphs
Monae [243]	Integration	Unpaired omics	Graph autoencoder	2024	Learning cross‐omics interactions for imputation and integration
UnitedNet [244]	Integration	Single‐cell multiomics	Explainable multitask learning	2023	Group‐level embedding and interpretation of multiomics relevance
scTFBridge [245]	Integration	Single‐cell multiomics	Disentangled deep generative model	2025	Disentanglement of latent spaces to infer TF regulatory activities
SpatialGlue [248]	Integration	Spatial multiomics	GNN + dual‐attention	2024	Deciphering spatial domains via intra‐ and cross‐omics integration
SWITCH [249]	Integration	Spatial multiomics	Graph attention network (GAT)	2025	Cross‐modal translation of spatial multiomics data
stClinic [252]	Integration	Spatial multiomics/phenotype	Dynamic graph model	2025	Integration of multislice spatial data with clinical phenotypes to identify niches
IBSEP [257]	Integration	Bulk RNA + scRNA	Statistical framework	2025	Integrates bulk and scRNA‐seq data for prioritizing cell‐type‐specific eQTLs
EXPRESSO [258]	Integration	scRNA + GWAS	Transcriptome‐wide association study	2024	Identify cell type‐specific risk genes
STdGCN [260]	Integration	SRT + scRNA	Graph convolutional network	2024	Cell‐type deconvolution in spatial transcriptomics using scRNA‐seq references
ST‐deconv [261]	Integration	SRT	Contrastive learning + autoencoder	2025	Accurate deconvolution utilizing self‐encoding and spatial representation enhancement
SDePER [262]	Integration	ST/scRNA	Machine learning/Regression method	2024	Deconvolve ST data using reference scRNA‐seq data
EnDecon [263]	Integration	SRT	Ensemble learning deconvolution method	2023	Predicting the cell type composition of SRT data
STged [264]	Integration	SRT	Non‐negative least‐squares regression framework	2025	Inferring cell‐type‐specific gene expression within individual spots
MISO [265]	Integration	SRT + histopathology images	Graph‐based autoencoder	2025	Improve spatial clustering
GHIST [269]	Integration	SRT + histopathology images	Multitask learning	2025	Inferring SRT from histopathology image at single‐cell resolution
ROSIE [270]	Integration	Spatial proteomics + histopathology images	ConvNet	2025	Translate histopathology image into multiplex spatial proteomics images
HistoPlex [271]	Integration	Spatial proteomics + histopathology images	GAN	2025	Translate histopathology image into multiplex spatial proteomics images
GigaTIME [272]	Integration	Spatial proteomics + histopathology images	Nested U‐Net	2026	Translate histopathology image into multiplex spatial proteomics images

**TABLE 2:** Foundation models for single‐cell and spatial omics analysis.

Model name	Journal	Date	Data size	Omics types	Function	References
scBERT	Nature Machine Intelligence	September 2022	1.1 million cells	Single‐cell transcriptomics	Cell type annotation	[276]
scFoundation	Nature Methods	June 2024	50 million cells	Single‐cell transcriptomics	Cell type annotation, perturbation prediction, cell/gene embedding, drug response prediction, read depth enhancement, gene module, and network inference	[277]
GeneFormer	Nature	May 2023	30 million cells	Single‐cell transcriptomics	Cell type annotation, perturbation prediction, cell/gene embedding, gene module, and network inference	[278]
scGPT	Nature Methods	February 2024	33 million cells	Single‐cell transcriptomics	Cell type annotation, perturbation prediction, batch integration, multiomics integration, and GRN inference	[279]
Updated Geneformer	bioRxiv	August 2024	103 million cells	Single‐cell transcriptomics	Cell type annotation, perturbation prediction, cell/gene embedding, and gene module identification	[280]
scTranslator	Nature Biomedical Engineering	November 2025	/	Single‐cell transcriptomics, single‐cell proteomics	Inference of missing single‐cell proteomic data	[282]
EpiAgent	Nature Methods	September 2025	5 million cells	Single‐cell epigenomics	Feature extraction, cell type annotation, and data imputation	[283]
SCimilarity	Nature	November 2024	23.4 million cells	Single‐cell transcriptomics	Cell type annotation and cell search	[284]
CellWhisperer	Nature Biotechnology	November 2025	1 million cells	Single‐cell transcriptomics	Interactive single‐cell analysis	[285]
scGPT‐spatial	biorxiv	February 2025	30 million cells	Spatial transcriptomics	Cell type annotation and gene imputation	[286]
KRONOS	aixiv	June 2025	47 million image tiles	Spatial proteomics (images)	Cell type phenotyping, region classification, artifact detection, patient stratification, and spatial biomarker discovery	[287]
Nicheformer	Nature Methods	October 2025	110 million cells	Single‐cell transcriptomics, spatial transcriptomics	Cell embedding and niche prediction	[288]
scPretrain	Bioinformatics	January 2022	0.9 million cells	Single‐cell transcriptomics	Cell type annotation	[291]
tGPT	iScience	May 2023	22.3 million cells	Single‐cell transcriptomics	Cell embedding	[292]
CellLM	aixiv	June 2023	2 million cells	Single‐cell transcriptomics	Cell type annotation and drug response prediction	[293]
CellPLM	biorxiv	October 2023	11 million cells	Single‐cell transcriptomics	Cell type annotation, cell clustering, and gene imputation	[294]
scHyena	aixiv	October 2023	/	Single‐cell transcriptomics	Cell annotation and data imputation	[295]
scCLIP	NeurIPS	November 2023	0.37 million cells	Single‐cell transcriptomics, single‐cell epigenomics	Multiomics integration	[296]
SATURN	Nature Methods	February 2024	0.33 million cells	Single‐cell transcriptomics	Cross‐species integration	[297]
scmFormer	Advanced Science	March 2024	1.5 million cells	Single‐cell transcriptomics, proteomics, single‐cell epigenomics	Multiomics integration	[298]
LangCell	ICML	May 2024	27.5 million cells	Single‐cell transcriptomics	Cell type annotation and integration	[299]
Cell‐Graph Compass	National Science Review	June 2024	50 million cells	Single‐cell transcriptomics	Cell type annotation, batch integration, and perturbation prediction	[300]
UCE	NeurIPS	October 2024	36 million cells	Single‐cell transcriptomics	Cell embedding cross different species	[301]
GeneCompass	Cell Research	October 2024	126 million cells	Single‐cell transcriptomics	Cell type annotation, perturbation prediction, GRN inference, drug response prediction, and gene embedding	[302]
Cell2Sentence	ICML	October 2024	/	Single‐cell transcriptomics	Cell embedding	[303]
scLong	Nature Communications	February 2026	48 million cells	Single‐cell transcriptomics	Perturbation prediction, drug response prediction, and GRN inference	[304]
GENEPT	Nature Biomedical Engineering	December 2024	/	Single‐cell transcriptomics	Cell/gene embedding	[305]
scMulan	biorxiv	December 2024	10 million cells	Single‐cell transcriptomics	Generate designated pseudo transcriptomics, cell annotation, gene imputation, and GRN inference	[306]
GET	Nature	January 2025	1.3 million cells	Single‐cell epigenomics	GRN inference	[307]
scPRINT	Nature Communications	April 2025	50 million cells	Single‐cell transcriptomics, single‐cell epigenomics	GRN inference and cell embedding	[308]
CellFM	Nature Communications	May 2025	102 million cells	Single‐cell transcriptomics	Cell type annotation, spatial gene imputation, and cell embedding	[309]
scCello	NeurIPS	May 2025	22 million cells	Single‐cell transcriptomics	Cell type annotation, prediction of cell‐type‐specific marker genes, and drug response prediction	[310]
SToFM	ICML	July 2025	88 million cells	Spatial transcriptomics	Cell type annotation and tissue region semantic segmentation	[311]
BrainBeacon	biorxiv	July 2025	133 million cells	Spatial transcriptomics	Analysis of biological mechanisms	[312]
CELLama	Advanced Science	November 2025	/	Single‐cell transcriptomics, spatial transcriptomics	Cell embedding	[313]

### 3.1 Single‐Cell Data Analysis

#### 3.1.1 Single‐Cell Genome Analysis

Single‐cell genome analysis provides a direct view of genomic alterations at cellular resolution, for understanding clonal dynamics, genomic instability, and evolutionary trajectory, yet remains constrained by low sequencing coverage. This challenge has motivated the development of two major categories of methods. The first reconstructs copy‐number alterations (CNAs) directly from scWGS profiles. For example, HiScanner [145] enables CNA detection from shallow scDNA data, while SCICoNE reconstructs CNA histories in tumor samples using statistical modeling and MCMC sampling [146]. MEDALT further infers lineage trajectories to identify fitness‐associated alterations and genes [147].

A second group of methods infers SNPs or CNAs from nongenomic single‐cell modalities, including transcriptomic [148] and chromatin accessibility profiles [149]. SComatic [150] and Cellsnp‐lite [151] are common methods for detecting SNPs from scRNA‐seq or ATAC‐seq data. Approaches such as Numbat [148] and CopyKAT [152] yield better performance in extracting CNA‐like signals from scRNA‐seq data in a benchmarking study [153], with CopyVAE [154] extending this strategy using variational autoencoders to improve robustness.

As scDNA‐seq protocols continue to evolve but coverage remains limited, these inference and lineage‐reconstruction models provide practical strategies for characterizing genomic heterogeneity. Continued benchmarking and methodological refinement will be crucial for single‐cell genome analysis, especially for reliably reconstructing clonal evolution reliably at cellular resolution.

#### 3.1.2 Single‐Cell Transcriptome Analysis

Single‐cell transcriptome analysis has become the most extensively employed approach for dissecting cellular heterogeneity. A comprehensive ecosystem of software packages and pipelines, including Seurat [155], Scanpy [156], Bioconductor‐based workflows, and command‐line toolkits, provides end‐to‐end analysis of scRNA‐seq data, covering QC, normalization, dimensionality reduction, clustering, and visualization of scRNA‐seq data [157]. These frameworks have become standard platforms for single‐cell analysis and are continuously extended to support multimodal assays and large‐scale atlas datasets.

A central challenge in scRNA‐seq analysis is the high sparsity and noise of count matrices, caused by limited mRNA capture efficiency, amplification bias, and stochastic gene expression. Numerous methods have been proposed to denoise or impute scRNA‐seq data based on deep generative models and graph‐based modeling, such as SmartImpute [158], scVGAMF [159], DCA [160], and GraphSCI [161]. Deep generative models like DCA adopt variational autoencoder architectures with count‐based likelihoods (e.g., ZINB) to jointly model noise and biological variation. Meanwhile, GraphSCI [161] simultaneously learns gene–gene relationships and cell–cell correlations, enabling improved imputation by integrating gene co‐expression networks and cell‐specific expression profiling into a stacked graph convolutional networks (GCNs)‐autoencoder model. Besides, SAVER‐X [162] used a transfer learning scheme to learn transferable gene–gene relationships from different datasets to achieve data denoising for noisy scRNA‐seq data while realize ample usage of existing data.

Dimensionality reduction and clustering are crucial steps for identifying cell populations and transcriptional programs [163]. Classical methods such as PCA and graph‐based clustering have been complemented by deep learning and manifold‐learning algorithms, which can better capture continuous trajectories and non‐linear structures in the data. Methods such as scVI [164], VASC [165], scvis [166], and scDHA [167] used autoencoder‐based models to learn low‐dimensional representations that preserve complex manifolds while mitigating technical noise and batch effects. While traditional clustering methods cannot flexibly regulate the process to achieve optimal results, scDeepCluster [168] was proposed by applying an autoencoder model, which integrated clustering loss with ZINB loss to perform clustering, and scDSC [169] incorporated GNN‐based structural information among cells to improve clustering quality. Knowledge‐guided models such as d‐scIGM incorporated domain constraints to generate biologically meaningful clusters [170] and another unsupervised deep model jointly optimize clustering and batch correction [171].

Cell‐type annotation, which is traditionally performed by manually matching cluster‐specific markers to known cell types, is increasingly automated by supervised and reference‐based methods. Tools such as BERMUDA [172], scScope [173], MARS [174], and scArches [175] employed transfer learning or deep metric learning (DML) to map new datasets onto existing reference atlases while correcting for batch and platform variation. Specifically, scScope supported scalable analysis of millions of noisy scRNA‐seq profiles and facilitates rapid identification of cell composition [173]. To discover novel cell types, MARS [174] used a deep learning scheme to learn from different datasets to perform cell‐type annotation on unannotated scRNA‐seq data. mtSC also leveraged multiple reference datasets by a multitask DML model to improve annotation reliability across studies [176]. While these methods are still time‐consuming and required reference datasets, GPTCelltype enabled efficient and automated cell type annotation using chain‐of‐thought prompt learning of GPT‐4 with comparable performance [177].

Trajectory and state‐transition inference remain essential for understanding dynamic biological processes. RNA velocity [178] and improved frameworks such as scVelo [179] estimated future transcriptional states by leveraging spliced and unspliced transcripts. For longer‐timescale developmental or lineage trajectories, probabilistic and deep‐learning approaches were developed. VITAE [180] combined a hierarchical mixture model with variational autoencoders to infer branching trajectories, whereas PRESCIENT [181] applied a deep generative framework to reconstruct cell‐state transitions and lineage structure. Besides, there are other tools for perturbation response prediction and disease state prediction for single‐cell data. For example, CellOT leveraged optimal transport theory and convex neural architectures to develop a framework for predicting the response of cells to given perturbation [182].

Some algorithms were designed to associate the scRNA‐seq data with phenotypic information. Scissor was developed by using the similarity between individual single cells and bulk samples as a constraint in a regression model to identify relevant subpopulations [183]. PENCIL was developed to perform simultaneous function of selecting informative features and identifying cell subpopulations by deploying a Learning with rejection strategy [184]. ScRAT applied attention‐based neural network to quantify the cell–cell interaction and the contributions of each cell in phenotype prediction [185]. scPhase utilized an attention‐based multiple instance learning to learn a patient‐level representation, which predicted clinical phenotypes across multiple patient cohorts [186].

Finally, quantum computing has been explored as an alternative computational paradigm. Quantum annealing strategies have been applied to optimize clustering cost functions [187], capture non‐linear gene interaction patterns for feature selection [188], and simulate gene regulatory interactions for network inference [189]. While these approaches are still exploratory, they suggest potential directions for future research alongside classical algorithms.

The wide range of computational methodologies developed for single‐cell transcriptome have not been expansively described in this section due to the limited scope. More detailed description for these methods can be referred to the specialized reviews [190, 191, 192].

#### 3.1.3 Single‐Cell Epigenome Analysis

Single‐cell epigenomic assays, including ATAC‐seq, DNA methylation, and single‐cell Hi‐C, capture chromatin accessibility, regulatory state, and 3D genome structure. Computational workflows typically involve peak calling or bin‐based quantification, followed by dimensionality reduction, clustering, and inference of regulatory motifs or transcription factor activity. As these datasets are markedly sparser and noisier than scRNA‐seq, their analysis requires methods tailored to modality‐specific sparsity and coverage constraints. EpiScanpy extended the Scanpy framework to single‐cell epigenomic data, addressing modality‐specific issues via multiple feature‐space constructions and nearest‐neighbor graph building [193]. For chromatin accessibility, SnapATAC supported large‐scale single‐cell ATAC‐seq analysis and trajectory reconstruction [194], and SnapATAC2 further generalized to multiple single‐cell modalities [195]. Benchmarking studies suggested strong performance of SnapATAC and SnapATAC2, particularly for large‐scale chromatin datasets with complex cell‐type structure [196].

Single‐cell Hi‐C data measure cell‐level 3D chromatin organization, contributing to the analysis of transcription process varying between different cell types. The imputation and embedding of single‐cell Hi‐C data is challenging as its 2D contact map adds complexity in data structure [197]. Studies have applied advanced deep learning methods in embedding single‐cell Hi‐C data. Higashi used hypergraph representation learning to capture the latent structure in 2D contact map [198], suggesting a practical tool for imputation and embedding of single‐cell Hi‐C data. scDEC‐Hi‐C combined a chromosome‐wise convolutional autoencoder with a deep embedding and clustering model to improve imputation and cell clustering [199]. Comparative analyses indicated that BandNorm, scHiCTools, Higashi, FastHigashi, and scVI‐3D all showed competitive performance in imputation and embedding tasks [200]. To address limited scDNA methylation data, scHiMe inferred methylation profiles from single‐cell Hi‐C using joint modeling [201]. Integrative modeling of multiple epigenomic modalities is an active area of research. Approaches such as ConvNet‐VAEs attempted to combine HM and chromatin accessibility data using one‐dimensional convolutional variational autoencoders [202]. Continued development is required to integrate diverse epigenomic modalities into unified regulatory representations.

#### 3.1.4 Single‐Cell Proteomics and Metabolomics Analysis

Single‐cell proteomics provides direct measurements of protein abundance and posttranslational states. Computational analysis of these data involves preprocessing (including imputation, transformation, and normalization), feature selection, dimensionality reduction, and clustering, often using graph‐based or density‐based algorithms adapted from scRNA‐seq analysis. Tools such as SCPline provide streamlined preprocessing pipelines tailored to low‐input proteomic datasets [203]. Deep learning frameworks, including scPROTEIN, used contrastive learning to derive robust embeddings from multiple single‐cell proteomics datasets [204]. PINNACLE used a geometric deep learning approach, which aimed to model protein interactions by generating context‐aware protein representations based on cell‐type information from scRNA‐seq data [205]. These methods offer potential for dissecting disease mechanisms and protein‐level regulation at cellular resolution.

SCM identifies the small molecules involved in the metabolic activity of cells, providing supplements for single‐cell proteomics. Methods such as SCMeTA provide preprocessing and analytical pipelines for MS‐based SCM data [206].

Given the limited availability of single‐cell proteomics and metabolomics data, several methods infer protein or metabolic states directly from scRNA‐seq. BABEL was among the first to perform cross‐modal translation between single‐cell omics using multiple autoencoders [207]. However, benchmarking suggests that TransferData in Seurat v3 and v4 often performs competitively for predicting surface protein levels from transcriptomes [208]. For metabolism, scMetabolism quantifies metabolic pathway activity from scRNA‐seq profiles, while GEFMAP used geometric deep learning to predict metabolic states from gene expression, enabling approximation of metabolomic profiles when direct assays are unavailable [209].

### 3.2 Spatial Data Analysis

#### 3.2.1 Spatial Genome and Spatial Epigenome Analysis

Spatial genomic analysis aims to reconstruct allele‐specific CNAs, subclonal structure, and tumor evolutionary dynamics directly from spatially resolved tissue sections. Although current spatial DNA assays remain limited by low coverage and technical constraints, several computational tools have attempted to infer CNAs from Spatially resolved transcriptomics (SRT) or imaging‐based genomic measurements. CalicoST reconstructed allele‐specific CNAs and spatially informed evolutionary trajectories by integrating spatial coordinates with transcript‐derived genomic signals [210]. SlideCNA applied expression‐aware spatial binning for overcoming sparsity limitations of CNAs data while maintaining spatial signal to recover CNA patterns and demonstrates its potential for spatial subclone detection [211]. However, these inference methods have not been fairly benchmarked, which requires further investigation.

Methods developed for spatial epigenome analysis are limited. Most of the analyses of spatial ATAC data are performed by methods in manipulating single‐cell ATAC data, including ArchR [212] and SnapATAC2 [195]. spaPeakVAE was extended from spaVAE, which captured spatial dependencies of signals and enabled effective analysis in multiple tasks [213]. The analysis of spatial epigenome is more common in integrative analysis of multiple spatial omics layers.

#### 3.2.2 Spatial Transcriptome Analysis

Spatial transcriptome analysis encompasses the computational workflows used to interpret spatially resolved gene expression profiles and to delineate tissue organization at high resolution. Standard analytical pipelines typically include data preprocessing, spatial feature construction by integrating gene expression with spot coordinates and histology, dimensionality reduction, and downstream tasks such as spatial domain identification, detection of spatially variable genes (SVGs), and inference of spatial trajectories or cell–cell interactions [214]. A number of general‐purpose tools, such as Seurat, Scanpy, Giotto, and Squidpy, provide integrated frameworks for preprocessing, visualization, and exploratory analysis of ST datasets.

Spatial domain identification is an essential task of ST analysis, aiming to organize molecular observations together with their spatial context into coherent tissue regions. Numerous specialized methods have been developed to enhance the resolution and biological interpretability of these spatial partitions, including BayesSpace [215], stLearn [216], SpaGCN [217], STAGATE [218], and GraphST [219]. BayesSpace applied a Bayesian statistical method that used spatial neighborhood information for clustering analysis [215]. stLearn learned robust spatial clusters of gene profiling based on a spatial graph‐based imputation method, and inferred spatial trajectory by diffusion pseudotime‐based method [216]. SpaGCN integrated gene expression, spatial proximity, and histology using GCNs to identify spatially enriched patterns [217]. STAGATE employed a graph attention autoencoder to learn latent spatial embeddings [218], whereas GraphST leveraged self‐supervised contrastive learning to derive highly discriminative spot representations [219]. Benchmarking studies across 13 methods and 34 datasets highlighted persistent challenges in detecting discontinuous spatial domains and regions with subtle transcriptional gradients [220]. Notably, algorithms were also developed to perform SVGs identification, among which SPARK‐X, a non‐parametric method for effectively detecting spatially expressed genes from large‐scale SRT data, outstood among all the benchmarking methods [221]. Moreover, integrating multi‐slice SRT enhanced the generation of biologically relevant domains by combining multi‐view graph network, contrastive learning, and attention mechanisms [222].

In subcellular‐resolution ST, cell segmentation is a prerequisite for cell‐level analyses. Methods such as SCS combined imaging data and sequencing information using transformer architectures [223], whereas Bering used graph deep learning to incorporate transcript colocalization patterns [224]. UCS integrated nuclei segmentation from staining images and spatial transcriptome data to define cell boundaries [225]. These methods have facilitated cell‐level analyses in subcellular ST.

A key objective of spatial omics is the characterization of cellular niches, the localized microenvironments that shape cell states, interactions, and tissue‐level functions. CellCharter applied Gaussian mixture modeling to identify, characterize, and compare cellular niches across spatial datasets [226]. scNiche resolved niches at single‐cell resolution by extracting multi‐view cellular features [227]. NicheCompass learned interpretable cell embeddings using graph‐based neural networks to map niche structure and organization [228]. Together, these approaches provided effective frameworks for dissecting microenvironmental architecture and its functional relevance within tissues. More detailed information about spatial transcriptome analysis methods can be referred to these specialized reviews [13, 229, 230, 231].

#### 3.2.3 Spatial Proteomics and Spatial Metabolomics Analysis

Spatial proteomics analysis aims to quantify cell phenotypes, signaling states, and microenvironmental organization from multiplexed protein measurements in tissue sections. Cell segmentation and spatial clustering are also important tasks in spatial proteomics analysis. For MSI data, HIT‐MAP [232] and SubCellBarCode [233] provided integrated pipelines for spatial proteomics analysis. For multiplexed IF (mIF) imaging data, AnnoSpat identified cell types and quantified cell–cell proximity relationships in spatial proteomics data [234]. STARLING applied a probabilistic machine learning model to quantify cell populations and cellular phenotypes from spatial proteomics data [235]. NPF was proposed to improve the resolution of sequencing‐based spatial proteomics methods [236]. Algorithms also enabled the connection of spatial proteomics with clinical phenotypes. Study has designed a geometric deep learning method and constructed spatial cellular‐graph to model tumour microenvironments from multiplexed spatial proteomics profiles [237], which facilitate spatial biomarker discovery.

Computational methods for spatial metabolomics are limited. SmartGate enabled iterative peak selection and spatial structure identification for imaging MS‐based spatial metabolomics data analysis [238]. MetaVision3D facilitated the transformation of serial 2D imaging sections of brain into 3D spatial metabolome with high resolution [239]. Moreover, these analyses are limited to single‐omics levels, while integrative analysis methods should be developed to gain biological insights.

### 3.3 Integrative Analysis

#### 3.3.1 Integrative Analysis Across Single‐Cell Multiomics Data

While the analysis of different single‐cell omics data provides different perspective of cellular activities, the integrative analysis of single‐cell multi‐omics data offers a more comprehensive picture of cellular states and regulatory programs. Many algorithms were developed to integrate single‐cell epigenome data with single‐cell transcriptome data, to reveal how regulation in chromatin and methylation links to RNA expression. Some earlier algorithms including MOFA+ [240] projected single‐cell multio‐mics data into a shared low‐dimensional latent space by matrix factorization to capture major sources of variation across modalities.

However, the integration of single‐cell multiomics data meets challenges. First, the datasets may not be measured in matched cells, bringing obstacles to single‐cell multi‐omics analysis. Some studies developed algorithms specific to the measurement of unpaired single‐cell multi‐omics data. UnpairReg performed regression analysis on unpaired single‐cell multio‐mics data by considering the biological associations between regulatory elements and between target genes, providing an accurate estimation of scRNA‐seq data where only scATAC‐seq data are available [241]. GLUE constructed modality‐guided regulatory graphs based on prior knowledge and aligns omics‐specific embeddings through adversarial learning, enabling cross‐omics regulatory inference, which even works for triple omics (RNA, ATAC, methylation) integration [242]. Monae designed multiple graph‐based autoencoders for learning cross‐omics interactions, facilitating imputation and integration of unpaired single‐cell multi‐omics data [243]. Recent work aimed to construct interpretable latent spaces by disentangling shared and modality‐specific components from single‐cell multio‐mics data. UnitedNet provided an explainable VAE‐based framework by grouping cell embeddings and performing contrastive learning on group‐level embeddings, facilitating the interpretation of group‐specific multi‐omics relevance, including RNA–protein correlation at single‐cell level [244]. scTFBridge has innovatively disentangled learned latent spaces of single‐cell multio‐mics data into shared and specific components, where the shared components are suggested to be related to specific TF regulatory activities [245]. While the complexity of single‐cell multi‐omics data requires further disentanglement, these methods have enriched the interpretation of complex molecular activities within biological systems, suggesting potential tools for biomedical discoveries. Additional methods for single‐cell multi‐omics analysis can be found in these reviews [246, 247].

#### 3.3.2 Integrative Analysis Across Spatial Multi‐omics Data

Integrative analysis of spatial multi‐omics data contributes to a more complete view of molecular regulation and biological pathways. SpatialGlue developed a graph neural network model with a dual‐attention mechanism that deciphers spatial domains by intraomics integration of spatial location and omics measurement followed by cross‐omics integration [248]. SWITCH is a deep generative model for cross‐modal translation of spatial multiomics data by graph attention networks (GATs) [249]. SpaFusion designed graph‐based autoencoders with high‐order cell graph of spatial transcriptome and spatial proteome as input to enable accurate clustering [250]. The model integrated multi‐level information, capturing both omic‐specific features and unified consensus representations across different omics data. The integration of ST and SM data meets challenges in different data distribution, while SpatialMETA constructed a conditional variational autoencoder to overcome the difficulties and to facilitate interpretation of spatially correlated ST–SM patterns [251].

In addition to improving the performance of common spatial omics tasks, spatial multi‐omics integration can also be used for downstream biomedical discovery. For example, stClinic designed a dynamic graph model that integrates spatial multi‐slice multiomics and clinical phenotype data to uncover clinically relevant niches [252]. Some other algorithms are developed to integrate spatial omics across different platforms [253] or different samples. PASTE provided cross‐slice alignment by Gromov–Wasserstein optimal transport algorithm [254]. CAST is a deep graph neural network‐based method for cross sample alignment of spatial omics at the single‐cell level [255]. These approaches lay the groundwork for comparative spatial analyses across tissues, conditions, and technologies. Moreover, additional representation strategies and comprehensive summaries are available in this recent review [256].

#### 3.3.3 Cross‐Scale and Cross‐Modal Integration

Integrative frameworks that link single‐cell, spatial, and bulk‐level omics enable cross‐scale characterization of molecular and cellular phenotypes in complex tissues. Among these integrative approaches, methods that characterize regulatory variation, especially single‐cell eQTL (sc‐eQTL) analyses, offer a way to resolve cell‐type specific genetic effects. For example, IBSEP improved cell‐type‐specific eQTL (ct‐eQTL) prioritization by combining bulk RNA‐seq and scRNA‐seq data [257]. EXPRESSO analyzed sc‐eQTL summary statistics by integrating 3D genomic data and epigenomic annotation to prioritize causal variants [258]. gsMap integrated ST data with Genome‐wide association studies (GWAS) summary statistics to map spatially resolved cell activity to human complex traits [259]. Together, these methods facilitated cross‐scale data analysis and provided insights for the underlying mechanism of human complex traits.

ST often suffers from limited resolution, as each spot typically captures transcripts from multiple cells. To address this, deconvolution methods aim to infer cell‐type compositions and cell‐type specific expression profiles, by leveraging scRNA‐seq reference datasets. Representative methods, including RCTD, SPOTlight, cell2location, and Tangram have been systematically reviewed elsewhere [214]. More recently, graph‐based and deep learning models such as STdGCN, ST‐deconv, SDePER, and EnDecon extend these ideas by incorporating spatial neighborhood structure, contrastive representation learning, or ensemble strategies to improve accuracy and robustness in heterogeneous tissues [260, 261, 262, 263]. Meanwhile, STged moved beyond traditional deconvolution by reconstructing cell‐type‐specific expression profiles directly from mixed spots, enabling fine‐grained expression modeling from low‐resolution spatial data [264].

Cross‐modal integration of spatial omics and histopathology images is another important direction. Some studies are integrating spatial omics and histopathology images to improve spatial domain identification, including MISO [265], SpaCell [266], and StereoMM [267]. Typically, MISO extracted graphs from spatial multi‐omics and histopathology image, and designed autoencoders for interpretable spatial clustering. Other studies performed cross‐modal prediction from histopathology images to spatial profilings. HistoST and THItoGene applied transformer framework for extracting morphology features from histopathology images and generate spatial transcriptomes, while BLEEP and mclSTExp performed contrastive learning for aligning the histopathology image with spatial transcriptome data [268]. The prediction can also reach single‐cell level [269]. ROSIE performed translation of histopathology to mIF data using ConvNet framework [270]. HistoPlex designed a GAN network to translate histopathology image into multiplex spatial proteomics images [271]. GigaTIME applied nested U‐Net framework to perform the similar task, which was validated on 14,256 patients with only histology data to generate virtual mIF data [272]. These cross‐modal inference methods not only enhanced the understandings of genotype‐phenotype associations, but also provided potential tools for clinical translation of spatial omics.

### 3.4 Foundation Models for Single‐Cell and Spatial Omics

Single‐cell and spatial omics datasets are high‐dimensional and noisy, making them well suited to representation learning. Many AI‐based analysis methods rely on transformers, graph neural networks, generative adversarial networks, or variational autoencoders to derive cell‐ and gene‐level embeddings [273, 274, 275]. Foundation models extend this idea by pretraining on large unlabeled datasets and then adapting to downstream tasks, potentially offering reusable representations across studies [144]. These methods not only facilitate efficient analysis of data, but also enable downstream analysis including clinical applications.

For single‐cell omics, several transformer‐based foundation models have been proposed, including scBERT [276], scFoundation [277], GeneFormer [278], and scGPT [279]. These models have shown improved performance in tasks such as clustering and batch correction compared with conventional methods like Seurat. Among the models, scGPT was able to perform single‐cell multi‐omics integration, and the updated version of GeneFormer [280] was pretrained on the largest single‐cell datasets. Single‐cell foundation models are suggested to be robust tools for diverse applications, while machine learning models are efficiently adapted to specific datasets [281]. scTranslator facilitated the cross‐modal translation from single‐cell transcriptome to single‐cell proteome by pretraining on large‐scale single‐cell multi‐omics data, yielding better performance than previous methods [282]. Epiagent [283] is a foundation model for single‐cell epigenome analysis. SCimilarity [284] facilitated scalable search for similar cells by metric learning and pretraining on single‐cell atlas datasets. CellWhisperer [285] enabled interactive single‐cell data exploration based on dialogues. Besides, agents for semi‐automated analysis pipelines are also emerging.

Foundation models on spatial omics are still limited. scGPT‐spatial [286] presented a foundation model for ST, which was designed to enhance SRT data analysis through continual pretraining of scGPT. KRONOS is a foundation model built for spatial proteomics, which was trained in a self‐supervised way on over 47 million image patches covering 175 protein markers, 16 tissue types, and 8 imaging platforms [287] and achieved state‐of‐the‐art performance across cell phenotyping, treatment response prediction, and retrieval tasks. Nicheformer learned cell representations that captures spatial context from spatial and single‐cell omics data based on a transformer framework, allowing the transfer of rich spatial information to scRNA‐seq data and the characterization of multi‐scale spatial niche for disease mechanism analysis [288]. OmiCLIP [289] enabled the cross‐modal inference of spatial transcriptome from histopathology images. Recently, the development of SpatialAgent [290] integrated large language models to form agent tools, which facilitated the automatic investigation of spatial omics data and experimental preparation, which innovating the spatial biology investigation.

Overall, foundation models offer a promising direction for standardizing representations and accelerating downstream analyses, but questions remain regarding data curation, cross‐cohort generalizability, interpretability, and fair evaluation across tasks and platforms.

## 4 The Application of Single‐Cell and Spatial Omics in Human Biology and Disease

**FIGURE 4:**
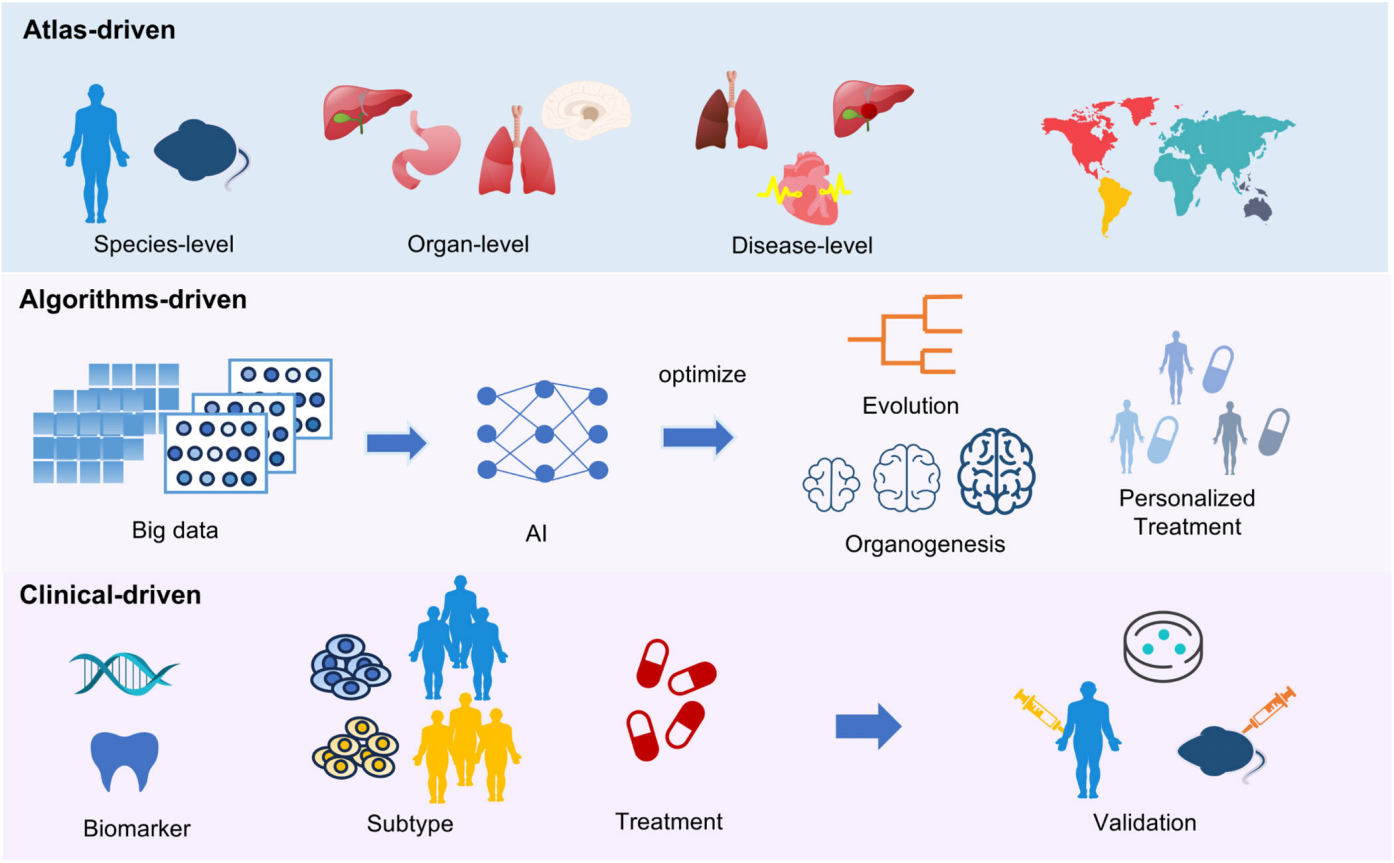
Applications of single‐cell and spatial omics analysis in biology and disease. The applications of single‐cell and spatial omics analysis in biology and disease can be classified into three types, including atlas driven, algorithm driven, and clinical driven. Constructing atlases at different levels provided a foundational map for biology and medicine. Developing algorithms specific to biomedical problems enabled AI‐driven biomedical discovery. Analysis according to clinical need facilitated the biomarker discovery, subtype identification, and drug discovery, some of which were validated by preclinical experiments and clinical trials.

Single‐cell and spatial omics have contributed to multiple aspects of applications in human biology and disease, ranging from developmental biology, evolutionary biology, disease mechanism identification, and clinical translation. These applications are divided into atlas‐based, algorithms‐based, and clinical translation‐based, which are summarized in the following subsections and Figure 4.

### 4.1 Constructing Data Cohorts and Single‐Cell Spatial Atlas for Biomedical Analysis

Single‐cell and spatial atlases constitute a foundational resource for biomedical discovery, providing reference maps of human cell types, tissue architecture, and disease‐associated cellular ecosystems. These resources span species‐level, organ‐level, disease‐level, and longitudinal datasets, collectively enabling systematic characterization of human biology in both health and disease (Table 3).

**TABLE 3:** Selected single‐cell/spatial atlas for all organs and systems.

System	Organ/tissue	Species	Atlas name^a^	Omics types	Healthy/diseased	Link
Multisystem/whole‐body	Multiorgan	Human	[Human cell atlas [314]]	Single‐cell transcriptomics	Healthy + diseased	https://data.humancellatlas.org/
	Multiorgan	Human	[Single cell atlas [315]]	Single‐cell transcriptomics	Healthy	http://www.singlecellatlas.org/
	Multiorgan	Human	[Tabula sapiens [316]]	Single‐cell transcriptomics	Healthy	https://tabula‐sapiens‐portal.ds.czbiohub.org/
Nervous system	Brain	Human/mouse	[Brain cell atlas [317]]	Single‐cell transcriptomics, spatial transcriptomics	Healthy	https://www.braincellatlas.org/dataSet
	Brain	Human	[Multiregion atlas of Alzheimer's disease [334]]	Single‐cell transcriptomics	Alzheimer's disease	https://www.synapse.org/#!Synapse:syn31512863
	Brain	Human	[Spatial proteomics and transcriptomics of glioblastoma [341]]	Spatial proteomics and transcriptomics	Glioblastoma	https://www.ncbi.nlm.nih.gov/geo/query/acc.cgi?acc=GSE237183
Respiratory system	Lung	Human	[Lung cell atlas [320]]	Single‐cell transcriptomics	Healthy + diseased	https://cellxgene.cziscience.com/collections/6f6d381a‐7701‐4781‐935c‐db10d30de293
	Bronchial/lung/BALF	Human	[COVID‐19 immune atlas [329]]	Single‐cell transcriptomics	COVID‐19	https://ngdc.cncb.ac.cn/gsa‐human/browse/HRA001149
	Lung/BALF	Human	[Bronchoalveolar immune atlas [330]]	Single‐cell transcriptomics	COVID‐19	https://www.ncbi.nlm.nih.gov/geo/query/acc.cgi?acc=GSE171524
	Lung	Human	[Lethal lung atlas [331]]	Single‐cell transcriptomics	COVID‐19	https://duos.broadinstitute.org
	Lung	Human	[Posttuberculosis lung atlas [347]]	Single‐cell transcriptomics	Tuberculosis	https://ngdc.cncb.ac.cn/gsa‐human/
Cardiovascular system	Left ventricle	Human	[Dilated and hypertrophic cardiomyopathy atlas [349]]	snRNA‐seq, bulk RNA	DCM/HCM	https://singlecell.broadinstitute.org/single_cell
	Heart	Human	[Atherosclerotic plaque spatial atlas [350]]	Spatial transcriptomics	Atherosclerosis	https://www.cncb.ac.cn/search?dbId=&q=HRA006030
Digestive system	Intestine	Human	[Multimodal spatial cell atlas of intestine [319]]	Single‐cell transcriptomics, spatial transcriptomics	Healthy	https://portal.hubmapconsortium.org/
	Pancreas	Human	[PDAC atlas [323]]	Single‐cell transcriptomics	Pancreatic cancer	https://ngdc.cncb.ac.cn/gsa/browse/CRA001160
	Liver	Human	[Spatial atlas of liver cancer [338]]	Spatial transcriptomics	Liver cancer	https://db.cngb.org/data_resources/project/CNP0002199/
	Esophagus	Human	[Evolutionary atlas of ESCC [340]]	Single‐cell transcriptomics, spatial transcriptomics	Esophageal cancer	https://github.com/PansccSpat/SpatialMapESCC
Urinary system	Kidney	Human	[Multimodal spatial atlas of kidney [318]]	Single‐cell transcriptomics, spatial transcriptomics	Healthy	https://atlas.kpmp.org/explorer/
	Kidney	Cross‐species	[Single‐cell kidney atlas [322]]	Single‐cell transcriptomics	Healthy + diseased	https://www.kpmp.org
Hematopoietic and immune systems	Bone marrow	Human	[Pediatric AML longitudinal atlas [345]]	Single‐cell transcriptomics	Healthy+AML	https://ega‐archive.org/
	Multiorgan/cancer	Human	[Pan‐cancer T‐cell atlas [324]]	Single‐cell transcriptomics	Pan‐cancer	http://cancer‐pku.cn:3838/PanC_T
	Multiorgan/cancer	Human	[Pan‐cancer NK‐cell atlas [325]]	Single‐cell transcriptomics	Pan‐cancer	http://www.ncbi.nlm.nih.gov/geo/query/acc.cgi?acc=GSE212890
	Multiorgan/cancer	Human	[Pan‐cancer B cell atlas [326]]	Single‐cell transcriptomics	Pan‐cancer	http://www.ncbi.nlm.nih.gov/geo/query/acc.cgi?acc=GSE233236
	Multiorgan/cancer	Human	[Curated cancer cell atlas [327]]	Single‐cell transcriptomics	Pan‐cancer	https://www.weizmann.ac.il/sites/3CA
Reproductive and developmental systems	Placenta	Human	[SARS‐CoV‐2 placental niches atlas [344]]	Single‐cell transcriptomics, spatial transcriptomics	SARS‐CoV‐2 infection of the placenta	https://www.ncbi.nlm.nih.gov/geo/query/acc.cgi?acc=GSE222987
Skin and connective tissue	Lung/intestine/skin	Human	[Cross‐tissue fibroblast atlas [337]]	scRNA‐seq	Chronic inflammation	https://pmc.ncbi.nlm.nih.gov/articles/PMC9271637/

^a^ *Note*: Some of the atlases were named based on their description in the corresponding references.

#### 4.1.1 Species‐Level and Organ‐Level Reference Atlas

In 2016, the launch of The Human Cell Atlas (HCA) opened up the analysis of human biology at the single‐cell level. The current version of HCA project has constructed a single‐cell dataset covering 62 million cells from around 9100 human donors, providing a comprehensive reference for human cell biology investigation [314]. Complementing HCA, Single Cell Atlas [315] integrated eight kinds of multiomics data, including single‐cell and spatial omics across different healthy human tissues. The Tabula Sapiens constructed a multiple‐organ single‐cell transcriptomic atlas of humans, which identified nearly 500 types of cells [316]. These species‐level single‐cell atlases serve for a foundation that would facilitate disease mechanism discovery and treatment design.

An organ is a structural unit composed of multiple tissues capable of performing specific physiological functions, characterized by specialized cell types, defined size, and complex morphological structures (e.g., buds, tubes, branches). Organ‐level single‐cell or spatial atlas facilitated the understanding of organ development and function in fine‐grained resolution, and identified key cellular components during disease development process. The integration of single‐cell atlas data from organ‐level depicted the cell interactions in brain, kidney, and lung, among others. The Brain Cell Atlas [317] integrated human and mouse single‐cell transcriptomic data to chart regional brain development and neuronal diversity. Multimodal spatial cell atlas of human kidneys [318] and intestine [319] are as part of the Human BioMolecular Atlas Program (HuBMAP), which serve as reference for understanding the organs. Human Lung Cell Atlas was constructed on over 2.4 million cells from 486 individuals with healthy or diseased status, which identified shared cell states across multiple lung diseases [320]. Single‐cell and spatial transcriptome atlas of eight regions of human heart revealed cellular niches and potential drug targets for human cardiac [321]. Moreover, kidney atlases comparing human and mouse highlighted conserved pathways and species‐specific disease mechanisms [322].

Collectively, these reference atlases standardize annotation frameworks, support evolutionary and developmental analyses, and serve as essential baselines for disease‐focused investigations.

#### 4.1.2 Disease‐Level Data Cohorts and Single‐Cell Spatial Atlas

Disease‐focused atlases extend these foundations to pathological contexts. Many studies constructed single‐cell atlas by performing single‐cell sequencing on clinically diagnosed patient samples to reveal the disease mechanisms, especially for cancer. Early disease atlases were limited in scale due to technological and cost barriers, such as a Pancreatic ductal adenocarcinomas (PDAC) atlas profiling 57,530 cells from 35 tumors and controls, which revealed extensive intratumoral heterogeneity [323]. With technological maturation, cohort sizes have expanded dramatically, enabling cellular‐resolution characterization across hundreds of patient samples. Pan‐cancer single‐cell atlases encompassing more than 700 cases have delineated conserved and divergent immune, stromal, and malignant programs across tumors, identifying recurrent T cell exhaustion states [324], natural killer cell phenotypes [325], and cancer‐associated B cell populations [326]. Gavish et al. integrated scRNA‐seq data of 1163 tumour samples covering 24 cancer types to construct a pan‐cancer single‐cell transcriptome atlas, identifying 11 intratumor transcriptomic patterns [327]. Moreover, the analyses of disease biology mechanism have been deepened by collecting samples across different locations, tissues, stages, and clinical phenotypes. A study analyzed the single‐cell transcriptome data from different sites of collected 10 HCC patients with primary and/or metastatic tumors samples [328], which identified microenvironment‐based subtypes for predicting the prognosis of HCC patients. These resources collectively reshape our understanding of tumor microenvironment composition and intercellular communication.

Beyond cancer, disease‐level single‐cell atlases have illuminated key mechanisms in infectious and degenerative diseases. During the COVID‐19 pandemic, large‐scale single‐cell atlases of patient lungs and bronchoalveolar immune cells revealed the immune landscape and molecular pathology of SARS‐CoV‐2 infection [329, 330, 331]. Integrating single‐cell transcriptomics with bulk RNA‐seq and proteomics further uncovered transcriptional regulators such as FOXO3A in COVID‐19‐induced fibrosis [332]. For neurodegenerative diseases, single‐cell cohort studies have similarly provided population‐level insights into Alzheimer's disease (AD). A single‐nucleus transcriptomic atlas of the brain vasculature identified diverse cellular mediators that contribute to AD risk [333]. A multiregion atlas across 48 individuals with and without AD further delineated region‐specific vulnerable cell states associated with AD pathology [334]. In a larger cohort of 437 aged individuals, a comprehensive snRNA‐seq atlas of the prefrontal cortex revealed two major cellular community trajectories distinguishing progression toward AD dementia from normative aging [335]. Complementary single‐cell QTL analyses identified cell‐subtype specific regulatory variants and causal genes underlying AD susceptibility [336]. Moreover, cross‐tissue single‐cell transcriptomic atlases have also uncovered common pathogenic mechanisms shared across distinct diseases. In particular, conserved fibroblast phenotypes have been identified across inflammatory conditions such as inflammatory bowel disease (IBD), rheumatoid arthritis (RA), and pulmonary fibrosis, highlighting shared cellular programs across tissues [337]. These disease‐level single‐cell atlases elucidate molecular circuits, immune dysregulation, and microenvironmental remodeling underlying diverse pathologies.

Spatial omics provides anatomical context by localizing cell states and interactions within tissue microenvironments. Integrative single‐cell/spatial analyses have mapped disease‐specific niches, such as invasive zones in liver cancer [338], and spatially recurrent malignant states across pan‐cancer cohorts [339], illustrating how spatial architecture shapes tumor progression. Moreover, Chang et al. combined ST with scRNA‐seq to construct a multistage spatial evolution map of esophageal squamous cell carcinoma (ESCC), showing that cellular heterogeneity increased progressively and that invasive cells gradually breached the epithelial–mesenchymal interface to form a heterogeneous spatial landscape [340]. In glioblastoma, Greenwald et al. integrated ST and spatial proteomics to define 14 spatial modules (including eight malignant cell states) and confirming the coexistence of structured and unordered regions within the tumor [341]. Likewise, multimodal integration of single‐cell and ST profiling in metastatic breast cancer delineated three distinct spatial phenotypes across diverse clinicopathological backgrounds [342]. Integrative ST and scRNA‐seq also identified pathologic niches in idiopathic pulmonary fibrosis (IPF) [343] and SARS‐CoV‐2‐associated niches in the human placenta [344].

Longitudinal data construction and single‐cell atlas depiction identified dynamic molecule changes along disease progression or therapeutic interventions. A longitudinal single‐cell atlas of pediatric acute myeloid leukemia profiled diagnosis, remission, and relapse trajectories, mapping shifting cellular hierarchies [345]. Spatial proteomic profiling before and after glioma treatment revealed remodeling of tumor‐specific neoantigen regions and immune infiltration, providing direct evidence for evaluating the reshaping effect of treatment on the tumor microenvironment (TME) [346]. Furthermore, longitudinal single‐cell multiomics atlases characterized patients with COVID‐19 and tuberculosis, identifying temporal molecular changes associated with severity and recovery [347]. Cardiovascular atlases further demonstrated how cardiac cell states and intercellular communication remodel during heart failure or injury [348, 349, 350].

Together, these multiscale disease cohorts provide dynamic, single‐cell and spatially resolved reference frameworks that illuminate pathological mechanisms and establish essential resources for downstream computational modeling and translational research.

### 4.2 Developing Single‐Cell and Spatial Algorithms for Biomedical Discovery

While numerous algorithms have been developed to address general challenges in single‐cell and spatial omics analysis (summarized in Section 3), a subset of computational methods has been specifically designed to tackle biological and disease‐related questions. These specialized algorithms exploit intrinsic data properties and bridge the gap between methodological innovation and biomedical discovery, enabling insights that are directly relevant to understanding physiology, evolution, and pathology.

#### 4.2.1 Algorithms for Evolutionary and Developmental Biology

Understanding the evolution of life, encompassing both interspecies and cross‐species dynamics, has long been a research focus in biology. Recent advances in single‐cell omics have facilitated the construction of cross‐species cellular atlases, revealing both conserved and divergent regulatory landscapes across organisms. For instance, GeneCompass, leveraging 120 million human and mouse single‐cell transcriptomes, uncovered conserved gene regulatory mechanisms between these species [302]. Similarly, TranscriptFormer, an AI‐driven virtual cell model trained on single‐cell data from twelve species spanning 1.5 billion years of evolutionary history, provided a scalable computational platform for systematic cross‐species evolutionary studies [351]. Together, these approaches highlight how large‐scale single‐cell datasets combined with evolutionary modeling can illuminate fundamental principles of life history and organismal diversification.

In developmental biology, spatiotemporal single‐cell and spatial omics have become essential for reconstructing embryogenesis and organogenesis with cellular precision. A key analytical challenge lies in aligning spatial datasets across developmental time and integrating 3D morphodynamic information [352]. Spateo addressed this by enabling 3D reconstruction and digitization of whole‐embryo ST, revealing spatial gradients and signaling networks that drive mouse embryogenesis and asymmetrical cardiac organogenesis [353]. By integrating multisample ST data, Stereopy provided a general framework to dissect spatiotemporal cellular heterogeneity and niche‐mediated regulation, exemplified by studies of cortical and cardiac development [354].

Aging represents another biological process with inherent evolutionary significance. Single‐cell omics allows for the high‐resolution dissection of cellular and molecular changes associated with aging. Based on constructed aging clocks, Tarkhov et al. revealed that epigenetic aging is driven by both stochastic and coregulated changes through scDNA methylation and transcriptome joint analysis [355]. Single‐cell transcriptome‐based aging clocks can also reveal organ‐level aging properties. Matthew et al. revealed that heterochronic parabiosis and exercise reverse aging process in neurogenic regions with different ways [356]. Aging also characterizes the heterogeneity of human immune system function. The constructed sc‐ImmuAging based on the single‐cell transcriptome profiles of peripheral blood mononuclear cells has revealed interindividual heterogeneity during infection and vaccination [357].

Collectively, these tools exemplify how single‐cell and spatial algorithms can quantify evolutionary and developmental processes at unprecedented resolution.

#### 4.2.2 Algorithms for Disease Mechanism and Clinical Translation

Deciphering disease mechanisms and translating single‐cell and spatial discoveries into clinically meaningful insights have become major drivers of computational innovation. A growing class of algorithms now connect the single‐cell or spatial profiles with clinical phenotypes, enabling the identification of phenotype‐specific molecular patterns for diseases. Methods such as Scissor [183], PENCIL [184], and ScRAT [185] enabled the discovery of cell subpopulations specific to disease prognosis and classification, including the COVID‐19 severity‐specific biomarkers and immunotherapy response‐specific cell subpopulations. Bulk and single‐cell integrative tools such as scBGDL extended these capabilities by predicting clinical subtypes and risk groups from heterogeneous data, while identifying candidate driver genes underlying these classifications [358]. SingleDeep enabled disease classification across diverse conditions, including lupus, AD, and COVID‐19 [359]. Cancer‐Finder performed accurate cancer cell annotation on single‐cell and ST, identifying gene programs enriched at the tumor–normal interface associated with patient outcome [360]. Moreover, in spatial proteomics, geometric deep learning approaches uncovered spatial motifs within the tumor microenvironment that stratify cancer prognosis [237]. Spatial multiomics derived immune scoring systems further demonstrated the translational potential of these approaches, providing robust predictors of hepatocellular carcinoma recurrence [361].HEX provided a deep learning model to generate spatial proteomics from H&E images, and by combining original H&E image with virtual spatial proteomics for patient outcome prediction, the method identified spatial niches for therapeutic response prediction of lung cancer in a low‐cost way [362].

Individualized treatment was also facilitated by emerging single‐cell algorithms. Computational frameworks were developed to optimize personalized combination therapy design [363], prioritize multitargeting treatment for coinhibition of malignant clones [364], and predict patient‐specific responses to treatment [365]. Multiscale PHATE extracted multimodal cellular signatures predictive of clinical outcome, as shown in COVID‐19 datasets [366]. Evolutionary modeling tools reveal dynamic clonal behavior during disease progression. For example, SPRINTER [367] revealed widespread clone proliferation heterogeneity when applied to a longitudinal, primary‐metastasis‐matched dataset of non‐small cell lung cancer (NSCLC), while SCEVAN [368] reconstructed clonal substructure and spatial evolutionary trajectories in brain tumors. An emerging frontier involves quantifying causal effects and treatment perturbations directly at single‐cell resolution. Perturbation‐aware models such as CINEMA‐OT employed causal optimal transport to reveal mechanisms of environmental exposure, for example, diminished antiviral responses following cigarette‐smoke stimulation [369]. scCausalVI employed causality‐aware generative modeling to identify treatment‐responsive cell populations and their underlying molecular programs in infectious disease [370].

Together, these computational frameworks link single‐cell and spatial data to concrete biomedical applications, spanning fundamental biology to clinical translation.

### 4.3 Toward Clinical Translation and Precision Medicine

Single‐cell and spatial omics have facilitated the identification of cellular states, disease‐associated niches, and molecular circuits that directly inform clinical decision‐making. By linking high‐resolution molecular phenotypes to patient‐level phenotypes and outcomes, these approaches enable biomarker discovery, precise disease classification, therapy response prediction, and personalized treatment strategies across diverse disease contexts (Table 4). Beyond cancer and infectious diseases, recent studies across autoimmune disorders, cardiovascular disease, kidney disease, and chronic inflammatory conditions further demonstrate the translational potential of single‐cell and spatial omics profiling.

**TABLE 4:** Main discoveries of single‐cell and spatial omics for clinical translation.

Disease classification	Disease	Type	Discovery	References
Autoimmune disease	Ulcerative colitis	Biomarker	Inflammatory fibroblast populations predict disease severity and anti‐TNF therapy response.	[371]
	Crohn's disease	Biomarker	A pathogenic cellular module (GIMATS) was linked to resistance to anti‐TNF treatment.	[372]
	Systemic lupus erythematosus	Biomarker	Combined IFN and plasmablast signatures are associated with flare risk.	[373]
	Systemic lupus erythematosus	Subtype	Multiplexed single‐cell RNA sequencing (mux‐seq) identified two molecular subtypes for SLE.	[374]
Cancer	Colon cancer	Treatment validation	scRNA‐seq validated the efficacy of BRAF inhibitors and immunotherapy.	[375]
	Melanoma	Target	Malignant cell‐intrinsic programs guide novel therapy development.	[376]
	Multiple myeloma	Treatment validation	scRNA‐seq identified the efficacy of adjuvant multiple myeloma vaccines.	[377]
	Lung adenocarcinoma	Target	Apoptosis‐related gene STK24 identified as a therapeutic target.	[378]
	Non‐small cell lung cancer	Treatment validation	scRNA‐seq validated the efficacy of chemotherapy agents combined with immune‐checkpoint inhibitors.	[379]
	Thyroid micro‐papillary carcinoma	Biomarker	The PROS1–MERTK axis drives tumor progression and has been identified as a promising biomarker and therapeutic target.	[380]
	Pancreatic cancer	Target	Single‐cell analysis of circulating tumor cells identified driver gene mutations and predicted tumor neoantigens in a mouse model.	[381]
	Cutaneous T cell lymphomas (CTCL)	Biomarker	Topographical differences between immune cells and tumor cells predicted PD‐1 blockade response.	[382]
	Triple‐negative breast cancer	Biomarker	Fractions of proliferating CD8^+^TCF1^+^T cells and MHCII^+^ cancer cells predicted immune checkpoint blockade (ICB) response.	[383]
	Pan‐cancer	Subtyping	Spatial multiomics analysis revealed four distinct spatial CAF subtypes across cancer types.	[384]
	Liver cancer	Subtyping	Identified five immune subtypes: immune activation, immune suppression mediated by myeloid or stromal cells, immune exclusion, and immune residence phenotypes.	[385]
Cardiovascular disease	Heart failure	Biomarker	Single‐cell profiling of dilated cardiomyopathy and normal individuals identified disease‐associated cell states.	[386]
Infectious disease	RSV infection	Target	Single‐cell transcriptomic analysis of RSV infection identifies host factors and receptors as potential targets for antiviral intervention.	[387]
	SARS‐CoV‐2 infection	Target	Identification of multiple effective neutralizing antibodies from convalescent patients' B cells.	[388]
Others	Kidney diseases	Biomarker	A fibrotic microenvironment characterized by injured tubule cells predicts severe disease.	[389]
	Idiopathic pulmonary fibrosis (IPF)	Biomarker	Spatial omics identified distinct fibrotic niches in diseased lungs.	[390]
	Skin disease	Treatment	Treatment with JAK inhibitors (JAKi) for a lethal skin disease validated with in vivo experiment.	[391]

#### 4.3.1 Biomarker Discovery and Prognosis Prediction

Traditional bulk‐level biomarkers mask critical cellular heterogeneity, whereas single‐cell and spatial omics technologies can mine not only gene markers, but also cell states or spatial structural features for diagnosis and prognosis prediction or providing references for clinical treatment plan selection.

Single‐cell omics is an effective tool in identifying immuno‐oncology biomarkers for predicting the therapeutic response of cancers [392], suggesting important clinical values [393]. In cancer, spatial niches were identified as potential biomarkers for clinical diagnosis and prognosis. Chen et al. discovered an immune niche consisting of antitumoral macrophages, CD8^+^ T cells and natural killer T cells (MT^2^) for predicting survival in patients with small cell lung cancer [394]. Liu et al. revealed that the abundance of specific cancer‐associated fibroblast (CAF) subtypes correlates with patient survival in a pan‐cancer analysis, highlighting their potential as prognostic biomarkers [384]. Chang et al. identified a “CAF‐Epi” niche in ESCC for predicting the survival periods of patients in different risk groups [340]. Spatial biomarkers can also predict the immunotherapy response of cancer. For example, topographical differences between immune cells and tumor cells predicted PD‐1 blockade response of cutaneous T cell lymphomas (CTCL) [382], and fractions of proliferating CD8^+^TCF1^+^T cells and MHCII^+^ cancer cells distinguished the difference in immune checkpoint blockade (ICB) response of triple‐negative breast cancer [383]. These spatial level or single‐cell level information identified potential biomarkers for cancer, going beyond the molecule‐based biomarkers defined by bulk‐level omics. Furthermore, by predicting cell types from histopathology image and performing cell phenotyping analysis, study suggested the value of histopathology image as a cost‐effective alternative in characterizing tumor microenvironments and identifying biomarkers for immunotherapy response of NSCLC, which significantly motivated the clinical translation of single‐cell spatial biomarkers [395].

Beyond cancer, single‐cell or spatial biomarkers have shown strong clinical potential across diverse diseases. In IBD, inflammatory fibroblast populations predicted both disease severity and anti‐TNF therapy response [371], while a unique cellular module, GIMATS consisted of cell subpopulations was associated specifically with resistance to anti‐TNF treatment [372]. In systemic lupus erythematosus (SLE), the interferon signaling (IFN) signature and plasmablast signature were associated with flare risk [373]. In kidney diseases, the gene signatures of a fibrotic microenvironment characterized by injured tubule cells predicted severe disease status [389]. Similarly, in IPF, spatial omics identified distinct fibrotic niches in diseased lungs [390]. In cardiovascular disease, single‐cell profiling of dilated cardiomyopathy and normal individuals identified disease‐associated cell states, which are potential biomarkers for heart failure [386]. Single‐cell and spatial analyses also identified immune cell populations as important markers for chronic and infectious respiratory diseases [396].

Despite these advancements, the translation of single‐cell biomarkers into routine clinical assays requires standardization, large‐cohort validation, and platform harmonization. Nevertheless, these biomarkers establish a foundation for next‐generation diagnostics that incorporate cellular context and spatial architecture.

#### 4.3.2 Characterize Disease Subtypes and Guiding Personalized Treatment

By resolving cellular ecosystems and tissue architecture, single‐cell and spatial omics overcome the limitations of traditional bulk sequencing and enable precise differentiation of disease subtypes and tailored matching of treatment plans.

In cancer, this approach enabled the stratification of patients into different immune subtypes [385] and immune archetypes based on the composition of tumor immune microenvironment, guiding immunotherapy decision‐making. Single‐cell profiling also improved subtype classification in NSCLC [397], papillary thyroid carcinoma [398] and nasopharyngeal carcinoma [399], enabling tailored treatment strategies. Moreover, analysis of single‐cell transcriptome has identified ecotypes with different cellular states to stratify patients in breast cancer [400] and even pan‐cancer datasets [401].

Importantly, this paradigm extends to noncancer diseases, including autoimmune diseases. For example, multiplexed scRNA‐seq (mux‐seq) identified two molecular subtypes occupying different components of cell populations for SLE [374]. The accumulation of fragmented single‐cell studies and bulk omics studies further refined molecular subtypes for autoimmune diseases, which were discussed in this review [402]. These molecular subtypes are relevant to distinct treatment strategies, for example, corticosteroids would be a potential treatment for the neutrophil subtype of SLE, while the infliximab may target the inflammation subtype [402].

Spatial omics further refines subtype classification by mapping microenvironmental interactions. Spatial multiomics analysis revealed four distinct spatial CAF subtypes across cancer types and omics platforms, whose abundance and composition were associated with specific TME features and clinical outcomes [384]. Spatial omics analysis also provided evidence supporting existing cancer subtyping schemes. Some ST analysis identified the heterogeneity among histologic subtypes and molecular subtypes for cancer [403, 404], suggesting the potential role of spatial omics in refining molecular subtyping of cancer.

As a result, single‐cell and spatial omics provide actionable frameworks for patient stratification and personalized therapy selection across malignant, infectious, and autoimmune diseases.

#### 4.3.3 Treatment Development, Drug Discovery, and Clinical Trials

Single‐cell and spatial omics are accelerating therapeutic discovery by identifying targetable cell states, reconstructing drug–response landscapes, and enabling high‐resolution pharmacology.

The development of targets targeting abnormal activated pathways or mutated genes in tumor cells has high specificity with experimental validation. Gao et al. screened out tumor neoantigens by single‐cell analysis on circulating tumor cells, which can significantly inhibit the growth of mouse transplanted tumors [381]. Marks et al. discovered that specific signaling pathways were activated in drug‐resistant AML cells [405]. Targeting intercellular signaling pathways in the TME can shape TME for therapeutic values. Chen et al. discovered that specific signaling axes in PDAC promoted neural infiltration, and inhibiting this signaling axis could reduce the proportion of related cells and the rate of neural invasion [406]. By integrating GWAS and single‐cell transcriptome data, the apoptosis‐related gene STK24 was identified as a therapeutic target for lung adenocarcinoma [378]. Beyond cancer, this pattern was also extended to other diseases. For example, one study utilized spatial proteomics to identify JAK/STAT and IFN signaling pathways for driving a lethal skin disease, which was validated by in vivo experiments involving JAKi inhibition treatment on diseased mouse models [391].

Single‐cell and spatial omics technologies demonstrate unique advantages in deconstructing host immune responses to inform vaccine and therapeutic antibody development for infectious diseases. For instance, in RSV research, single‐cell transcriptomic profiling of infected cotton rats systematically mapped the landscape of host factors and inflammatory pathways involved in infection and recovery, providing a foundational resource for identifying novel targets for antiviral intervention [387]. In the study of SARS‐CoV‐2, researchers identified multiple effective neutralizing antibodies from a large number of antigen‐binding IgG1+ clonotypes by performing HT scRNA and VDJ sequencing on antigen‐enriched B cells from recovered patients [388].

Single‐cell and spatial omics analyses also played important roles in validation of treatment efficacy and biomarkers development in preclinical models and clinical trials (Table 5). Relevant clinical trials including observational, interventional Phase I and Phase II studies, have been employed to evaluate the efficacy of new therapies or identify cellular biomarkers for disease classification. Specifically, scRNA‐seq was used to identify changes in the immune microenvironment in patients following therapeutic interventions [17] and was applied to evaluate the efficacy of adjuvant multiple myeloma vaccines [377], BRAF inhibitors and immunotherapy for colon cancer [375], chemotherapy combined with immune‐checkpoint inhibitors for lung cancer [379], and neoadjuvant immunotherapy for cutaneous squamous cell carcinoma [407]. Beyond cancer, single‐cell TCR/BCR combined with scRNA‐seq also revealed treatment mechanism for autoimmune diseases, including RA [408] and autoimmune hemolytic anemia [409]. scRNA‐seq was also applied in observational clinical trials, where identified biomarkers for disease diagnosis [410] and treatment efficacy [411].

**TABLE 5:** Applications of single‐cell and spatial omics in preclinical animal experiments and clinical trials.

Trial type	No	Phase	Status	Disease	Sequencing technology	Therapies/diagnosis	Mechanism	References
Clinical trial	NCT03668431	Phase II	Completed	Colon cancer	scRNA‐seq	BRAF inhibitors and immunotherapy	Tumor cell‐intrinsic immune mechanism	[375]
Clinical trial	NCT02728102	Phase II	Completed	Multiple myeloma	scRNA‐seq	Adjuvant multiple myeloma vaccines	Clonotypic expansion of activated CD8 cells after vaccination	[377]
Clinical trial	NCT03158129	Phase II	Completed	Non‐small cell lung cancer	Single‐cell sequencing and multiplatform immune profiling	Neoadjuvant ipilimumab + nivolumab + chemotherapy (Ipi+Nivo+CT)	Increased effector memory CD8+ T, B and myeloid cells and markers of tertiary lymphoid structures	[379]
Clinical trial	NCT05878288	Phase II	Active, not recruiting	Cutaneous squamous cell carcinoma	scRNA‐seq; bulk genomic profiling	Neoadjuvant immunotherapy	/	[407]
Clinical trial	ACTRN12617001482358	Phase I	Completed	Rheumatoid arthritis	Single‐cell TCR/RNA‐seq	A novel nanoparticle‐based immunotherapy drug (DEN‐181)	T cell transcripts associated with tolerogenic TCR signaling and exhaustion after therapy	[408]
Clinical trial	NCT06231368	Phase I	Completed	Autoimmune hemolytic anemia (AIHA)	Single‐cell RNA/BCR‐seq	CD19 CAR T‐cell therapy	Crosstalk between HLA‐DRB5+ B cells, CD4+ T cells, and B‐cell maturation antigen‐expressing long‐lived plasma cells formed a B‐cell niche specific to relapse after therapy.	[409]
Clinical trial	NCT07081087	Observational	Recruiting	Cytopenia/myelodysplastic syndromes (MDS)	Single‐cell sequencing	Diagnosis and subclassification of MDS	/	[410]
Clinical trial	NCT04807114	Observational	Recruiting	Non‐small cell lung cancer	scRNA‐seq; scTCR‐seq; CITE‐seq	Immune checkpoint blockade	/	[411]
Preclinical experiment	/	/	/	Neuroblastoma	scRNA‐seq	Chemotherapy	Weak mesenchymal‐like gene expression programs after therapy	[412]
Preclinical experiment	/	/	/	Hepatocellular carcinoma	scRNA‐seq, tetramer flow cytometry, immunofluorescence	Personalized neoantigen vaccine combined with PD‐1 blockade	The increased percentage of CD8+ TRM was associated with antitumor efficacy.	[413]
Preclinical experiment	/	/	/	Multiple myeloma	scRNA‐seq	TIGIT inhibition to BCMA‐CAR‐T therapy	TIGIT modulates CAR‐T activity by regulating cytokines and chemokines pathways and T cell activation.	[415]
Preclinical experiment	/	/	/	Hepatocellular carcinoma	Spatial RNA‐seq; single‐cell RNA‐seq	Anti‐PD‐1 therapy	PLAUR+ neutrophils orchestrate immune evasion by CD8+ T cell exclusion and macrophage‐dependent immune suppressor.	[414]
Preclinical experiment	/	/	/	Adenomyosis	scRNA‐seq	Prolactin receptor	Prolactin (PRL) signaling as a key pathological driver of adenomyosis	[416]
Preclinical experiment	/	/	/	Primary sclerosing cholangitis	Single‐cell RNA‐seq; spatial RNA‐seq; multiplex proteomics	Claudin‐1	CLDN1‐specific monoclonal antibodies (mAbs) inhibited proinflammatory and profibrotic signaling in cholangiocytes and hepatocytes perturbed in PSC liver tissues.	[417]

In addition to these clinical trials, some studies remain at the preclinical stage. In preclinical animal models, analyses using scRNA‐seq, spatial RNA‐seq, and multiplex proteomics have identified potential mechanisms of disease treatment, including chemotherapy in neuroblastoma [412], anti‐PD‐1 therapy for HCC [413, 414], TIGIT inhibition combined with BCMA‐CAR‐T therapy in multiple myeloma [415], prolactin receptor‐targeted therapy in adenomyosis [416], and claudin‐1 based targeted therapy in primary sclerosing cholangitis [417].

While performing the preclinical animal experiments is laborious and costly, AI‐driven virtual cell was proposed as another avenue to intelligently perform preclinical testing and evaluation on drug response and patient progression [418]. The realization of this concept would greatly decrease the cost in experimental design and accelerate the clinical translation of novel treatments.

Moreover, ongoing efforts on developing algorithms to predict drug response from single‐cell profiles are promoting the drug discovery and screening process [182, 419, 420]. Technologies include multiplex scRNA‐seq pharmacotranscriptomics [421] and multimodal pharmacogenomics such as single‐cell EpiChem [422] have facilitated the measurement of drug–target engagement on single‐cell level. However, the role of spatial omics in drug discovery is still limited, which required further methodological developments.

Collectively, these advances position single‐cell and spatial omics as indispensable tools for therapeutic development, enabling rational drug design, combination therapy optimization, and precision pharmacology across a wide spectrum of diseases.

## 5 Challenges and Future Directions

Single‐cell and spatial omics have rapidly evolved into transformative technologies for dissecting developmental processes, tissue organization, and disease mechanisms at cellular and subcellular resolution. Despite these advances, several key challenges remain before these platforms can be deployed broadly across biological research and translational medicine.

Technological limitations remain a major bottleneck. Despite substantial improvements, current platforms still face constraints in cost, throughput, and comprehensive molecular detection. Long‐read technologies offer a promising avenue by enabling full‐length transcript and epitranscriptome profiling at single‐molecule resolution [423]. These capabilities are crucial for resolving RNA editing, isoform diversity, and regulatory variation in health and disease. Meanwhile, next‐generation spatial multiomics technologies must balance affordability with sensitivity; innovations such as low‐cost barcoding strategies (e.g., MiP‐seq) point toward more scalable platforms [424]. Continued development of integrative assays that coprofile genome, epigenome, transcriptome, and proteome within the same cell or tissue context will be essential for achieving a mechanistic understanding of molecular regulation. Equally important are emerging pharmacogenomics [425] and single‐cell pharmacotranscriptomics frameworks [421], which will facilitate cell‐type resolved drug discovery and accelerate precision therapeutics.

Methodological challenges persist on the computational side. Although a variety of computational algorithms and tools now exist for single‐cell and spatial data, including clustering, denoising, multimodal integration, spatial domain analysis, and phenotype prediction, their performance across diverse tissues, diseases, and experimental conditions remains uneven. Benchmarking studies often provide discordant recommendations, reflecting rapid algorithmic turnover and inconsistent evaluation standards [426]. The limited interpretability of deep learning‐based approaches further complicates biological insight. Recent advances in interpretable architectures have begun to distill biologically meaningful features from single‐cell and spatial gene profiles [427, 428], yet broader validation across disease systems is required. In the future, these methods should be applied on more datasets and diverse biomedical contexts to drive the biomedical discovery. Foundation models trained on massive single‐cell and spatial atlases introduce a powerful data‐driven paradigm by enabling unified embeddings, cross‐modal prediction, and improved generalization [144]. Quantum computing is also emerging as a potential future direction for spatiotemporal single‐cell analysis and cell‐based therapeutics [429], but its practical utility remains to be established. Future work, however, must determine how these models can be adapted and fine‐tuned for specific biological questions, disease settings, and clinical decision‐making. Importantly, algorithms on associating single‐cell or spatial profiles with disease or biology phenotypes have improved our understanding of genotype‐phenotype relationships and helped identify phenotypic‐specific molecular alterations. While more and more algorithms are developing, computational advancements in single‐cell and spatial omics analysis should serve for biomedical applications in the future.

Biological interpretation and translational application constitute the next frontier. Although extensive atlases have been generated across organs, developmental stages, and species, most atlas efforts remain dominated by transcriptomics, with limited incorporation of epigenomic, proteomic, metabolic, or functional perturbation data. Approaches such as single‐cell/spatial CRISPR screening provide mechanistic insight into genotype–phenotype relationships, enabling causal inference rather than correlation [430, 431, 432]. Future studies should focus on integration of existing atlas to enable cross‐organ, cross‐system, and cross‐species analyses, thereby generating a more comprehensive view of human biology system and providing insights for disease treatment. Translational application has lagged behind basic discovery; few molecular insights have progressed into clinical trials. More in‐depth analysis should be performed to identify effective biomarkers, disease subtypes and treatment strategies for registering clinical trials. Besides, histopathology image is becoming a promising cost‐effective alternative for spatial omics in some scenarios, including biomarker discovery, disease subtypes and even performing personalized treatment, which would accelerate the clinical translation of single‐cell omics or spatial omics [268]. However, methodological improvements and clinical cohort validation are required to further establish the reliability of histopathology as a surrogate for spatial omics. With the accumulation of data and methods, the development of cross‐scale multimodal foundation models is highly promising. These models can effectively link single‐cell spatial molecular information with variable clinical phenotypes, thereby providing high‐resolution disease genotype‐phenotype associations and promoting the development of personalized medicine.

Together, addressing these technological, computational, biological, and translational challenges will determine how single‐cell and spatial omics evolve from powerful discovery tools into foundational components of clinical research, precision diagnostics, and targeted therapeutics.

## 6 Conclusion

Recent years have witnessed major advances of single‐cell and spatial omics technologies and computational methods, which have greatly enriched the understanding of human biology and diseases of all organs and systems. Nevertheless, the successful integration of massive, heterogeneous cross‐modal datasets and the development of robust, scalable models to interpret them remain critical bottlenecks for the field. Future efforts on developing biology‐guided multiomics, cross‐modal sequencing technologies, and advanced data analysis are thus essential to provide in‐depth understandings of human biology at multiple resolutions, further accelerating clinical translation in personalized disease treatment.

## Author Contributions

Xiaoping Cen: data curation and writing – original draft. Xiaolan Huang: data curation and writing – original draft. Enjin Deng: data curation and writing – original draft. Xue Gong: investigation and writing – review and editing. Na Tan: investigation and writing – review and editing. Ye Jifeng: investigation. Yin Wang: writing – review and editing. Roland Eils: writing – review and editing. Qun Luo: writing – review and editing. Yixue Li: supervision, project administration, and funding acquisition. Fangfang Qu: supervision, conceptualization, writing – review and editing, and funding acquisition. All authors approved the final manuscript.

## Funding

This work was supported in part by the National Key R&D Program of China (2023YFF1204701 and 2025YFE0126600), Prevention and Control of Emerging and Major Infectious Diseases‐National Science and Technology Major Project (2025ZD01901900), National Natural Science Foundation of China (82400622 and 12371485), Guangdong Basic and Applied Basic Research Foundation (2025A1515011597), Major Project of Guangzhou National Laboratory (GZNL2025C01013 and SRPG22007), Young Scientists Program of Guangzhou Laboratory (QNPG23‐01), and Startup Program of Guangzhou National Laboratory (YW‐YFYJ0101).

## Ethics Statement

The authors have nothing to report.

## Conflicts of Interest

The authors declare no conflicts of interest.

## Data Availability

The authors have nothing to report.
